# Floral Assemblages and Patterns of Insect Herbivory during the Permian to Triassic of Northeastern Italy

**DOI:** 10.1371/journal.pone.0165205

**Published:** 2016-11-09

**Authors:** Conrad C. Labandeira, Evelyn Kustatscher, Torsten Wappler

**Affiliations:** 1 Department of Paleobiology, National Museum of Natural History, Smithsonian Institution, Washington, DC 20013–7012, United States of America; 2 Department of Entomology, University of Maryland, College Park, MD 20742, United States of America; 3 College of Life Sciences, Capital Normal University, Beijing, 100048, China; 4 Museum of Nature South Tyrol, Bindergasse 1, 39100 Bozen/Bolzano, Italy; 5 Department für Geo- und Umweltwissenschaften, Paläontologie und Geobiologie, Ludwig-Maximilians-Universität and Bayerische Staatssammlung für Paläontologie und Geobiologie, Richard-Wagner-Straße 10, 80333 München, Germany; 6 Steinmann Institute, University of Bonn, Nussallee 8, 53115 Bonn, Germany; Institute of Botany, CHINA

## Abstract

To discern the effect of the end-Permian (P-Tr) ecological crisis on land, interactions between plants and their insect herbivores were examined for four time intervals containing ten major floras from the Dolomites of northeastern Italy during a Permian–Triassic interval. These floras are: (i) the Kungurian Tregiovo Flora; (ii) the Wuchiapingian Bletterbach Flora; (iii) three Anisian floras; and (iv) five Ladinian floras. Derived plant–insect interactional data is based on 4242 plant specimens (1995 Permian, 2247 Triassic) allocated to 86 fossil taxa (32 Permian, 56 Triassic), representing lycophytes, sphenophytes, pteridophytes, pteridosperms, ginkgophytes, cycadophytes and coniferophytes from 37 million-year interval (23 m.yr. Permian, 14 m.yr. Triassic). Major Kungurian herbivorized plants were unaffiliated taxa and pteridosperms; later during the Wuchiapingian cycadophytes were predominantly consumed. For the Anisian, pteridosperms and cycadophytes were preferentially consumed, and subordinately pteridophytes, lycophytes and conifers. Ladinian herbivores overwhelming targeted pteridosperms and subordinately cycadophytes and conifers. Throughout the interval the percentage of insect-damaged leaves in bulk floras, as a proportion of total leaves examined, varied from 3.6% for the Kungurian (N = 464 leaves), 1.95% for the Wuchiapingian (N = 1531), 11.65% for the pooled Anisian (N = 1324), to 10.72% for the pooled Ladinian (N = 923), documenting an overall herbivory rise. The percentage of generalized consumption, equivalent to external foliage feeding, consistently exceeded the level of specialized consumption from internal feeding. Generalized damage ranged from 73.6% (Kungurian) of all feeding damage, to 79% (Wuchiapingian), 65.5% (pooled Anisian) and 73.2% (pooled Ladinian). Generalized-to-specialized ratios show minimal change through the interval, although herbivore component community structure (herbivore species feeding on a single plant-host species) increasingly was partitioned from Wuchiapingian to Ladinian. The Paleozoic plant with the richest herbivore component community, the coniferophyte *Pseudovoltzia liebeana*, harbored four damage types (DTs), whereas its Triassic parallel, the pteridosperm *Scytophyllum bergeri* housed 11 DTs, almost four times that of *P*. *liebeana*. Although generalized DTs of *P*. *liebeana* were similar to *S*. *bergeri*, there was expansion of Triassic specialized feeding types, including leaf mining. Permian–Triassic generalized herbivory remained relatively constant, but specialized herbivores more finely partitioned plant-host tissues via new feeding modes, especially in the Anisian. Insect-damaged leaf percentages for Dolomites Kungurian and Wuchiapingian floras were similar to those of lower Permian, north-central Texas, but only one-third that of southeastern Brazil. Global herbivore patterns for Early Triassic plant–insect interactions remain unknown.

## Introduction

The ecological crisis at the Permian–Triassic (P-Tr) boundary had a profound effect on life on the planet [1], and affected the terrestrial realm [2,3], just as it did for organisms living in marine environments [4]. Of the many consequences of this global ecological transformation were the effects on relationships among terrestrial organisms, such as plants and their associated arthropod herbivores [5–7], although much recent paleoecological work has focused on changes in vertebrate prey and their predator relationships before and after the P-Tr boundary [3,8]. Meanwhile, there also has been a parallel focus on understanding patterns of plant-host use by herbivorous and ovipositing insects [7,9,10] and terrestrial community structure at sites before [11–13] and after [8,14,15] the P-Tr boundary.

Studies of early (Cisuralian), middle (Guadalupian) and late (Lopingian) Permian age have involved field sites from a variety of tectonic and depositional settings that occur in regions often rich in terrestrial deposits [11,16,17]. Typically, stratigraphic sections from these areas coarsely sample a relatively narrow time interval within a Permian stage. These studies include Cisuralian localities that contain floras from which plant–insect associational quantitative data have been retrieved, primarily from north-central Texas of the United States [17–21], the northeastern part of the Paraná Basin in southeastern Brazil, and adjacent Argentina and Uruguay [22–27], although isolated basins along the eastern and western margins of the Andean Volcanic Arc in Argentina also have been explored [28]. Other inland basins from continental Gondwana, for which there is significant plant–insect interactional data, include the Karoo Basin of South Africa [11,29–32], Mohuda and Barakar basins of India [33–37] and the Bowen and Sydney basins of eastern Australia [31,38–41].

Permian plant–insect interactional data from Europe typically is older, generally anecdotal and has not involved quantitative analyses of bulk floras. These studies have focused on documentation and qualitative assessments of individual plant–insect relationships in a variety of depositional settings and ages. Detailed descriptions of associations have come from Western Europe [42–44], and from Russia in deposits associated with creation of the Ural Mountains and the Volga and Kama River basins [45–47]. One latest Permian site, the Sokovka locality in European Russia [48], records the earliest occurrence of leaf mining [45], a distinctive interaction whose earliest occurrence previously was recorded from the Middle Triassic [9]. In East Asia, interactions, including those of mite borings, have focused principally on lower Permian deposits in the Pingquan district of Hebei Province, North China [49], bored spores from the middle Permian Ningxia Hui Autonomous Region, North China [50], middle–upper Permian deposits of Taiyuan city and the Xiedao locality, both in Shanxi Province, North China [51,52], the Permian of the northern Helan Mountains, in the Inner Mongolia (Nei Mongol) and Ningxia Huizu Autonomous Regions, northern China [53] and from the upper Permian of Yunnan Province, South China [54], representing the North China and South China tectonic terranes, respectively.

All Triassic deposits that have been investigated in great detail currently lack quantitative data for plant–insect interactions. Even with considerable searching [9,45], the five-million-year time window of the Early Triassic has not produced floras from which any significant plant–insect associational data can be extracted, in spite of recent accounts that have revealed a few Early Triassic interactions along the P-Tr boundary interval in Europe [55] and Australia [31]. By contrast, Middle and Late Triassic sites have provided plant–insect interaction occurrences comparable in richness or exceeding those of the Permian. In Western Europe, particularly for deposits from France and Germany [14, 56–59], there is a modest record of plant–insect interactions in Middle and earlier Late Triassic deposits. For North America, the most notable locality is the Late Triassic Chinle Formation that has evidence for insect interactions on foliage from compression–impression material [60–62] and insect and mite wood borings within silicified trunks [63–65]. The Yipinglang Flora of the Chuxiong Prefecture in central Yunnan, China [66], also has provided Late Triassic data. From Gondwana, a significant amount of data has originated from several regional basins along the Andean Volcanic Arc in Argentina [25] and Chile [67,68], the extensive Late Triassic (Carnian) Karoo Basin of South Africa [9,69,70], and the Ipswich and Sydney basins of Eastern Australia [71,72]. These global occurrences, overwhelmingly are from the Middle and early Late Triassic, and indicate that Triassic plant–insect interactions are significantly undersampled.

Triassic studies have not been integrated with Permian studies that also examine plant–insect associations in long-term contexts involving tens of millions of years before and after the P-Tr boundary within the same basin or region. There are several reasons for this lack of continuity. First is the absence of relevant Triassic deposits that would locally correspond to those Permian deposits with significant plant–insect interactional data, or vice versa with Triassic deposits lacking significant Permian data equivalents. Second, is the lack of sufficiently resolved plant–host identifications. Third, is the poor preservational potential of likely sites that would disallow proper identification of insect-mediated damage. Last, there has been the historical absence of paleobiological interest [10].

**Fig 1:**
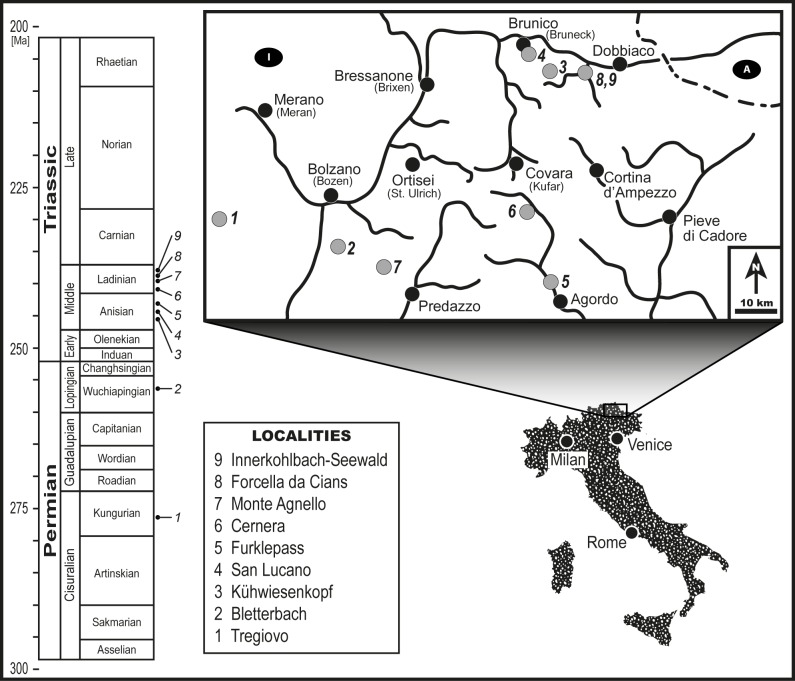
Stratigraphic and geographic context of localities that provide plant–insect interaction data for the Permian–Triassic sequence from the Dolomites Region in northeastern Italy. Geologic scale is after [76]; grey dots designate localities discussed in this report black dots refer to principal towns and villages; and lines delimit major roads.

In this contribution, we provide plant–insect associational data from a series of deposits in the Southern Alps of northeastern Italy–the Dolomites–that form a geographically confined but stratigraphically interrupted succession of deposits. The ten deposits in our study represent four general slices of time from the Kungurian of the late Cisuralian (Tregiovo locality); the Wuchiapingian of the early Lopingian (Bletterbach Gorge locality); the Anisian of the early Middle Triassic (the three localities of Kühwiesenkopf, Furkelpass and Valle San Lucano); and the Ladinian of the late Middle Triassic (the five localities of Monte Cernera, Monte Agnello, Forcella da Cians, Seewald and Innerkohlbach) (Fig 1). This study represents the first, systematic, synthetic compilation of Permian to Triassic plant–insect associational data for any stratal succession in Western Europe.

## Geological and Biological Setting

Several Permian to Triassic carbonate and terrigenous sequences crop out along the Dolomites Region of northeastern Italy. These outcrops document the episodic presence of floras along the southern rim of the Alps from late Paleozoic to early Mesozoic time. The strata of relevance for assessing the effects of the P-Tr ecological crisis in the Dolomites range from Kungurian to Ladinian in age. This temporally interrupted sequence documents the presence of floras that are sufficiently well preserved that richness assessments of the bulk floras and characterizations of plant–insect interactions can be made. The geological and biological contexts of the Kungurian, Wuchiapingian, Anisian and Ladinian floras are provided in the evaluation of herbivory that follows. Importantly, the Permian deposits establish a baseline to which comparisons of the Middle Triassic deposits can be assessed.

### Late Cisuralian (Kungurian)

#### Tregiovo Formation

Kungurian fossil plants come from the Le Fraine outcrop, near the village Tregiovo in the upper Val di Non area, in the Trentino–Alto Adige Region of Northern Italy (Fig 1; for additional details, see reference [13]). The compression and impression fossils were collected from the Tregiovo Formation, which was deposited as a small sedimentary basin about two km wide in the Athesian Volcanic District during a period of relative stasis within an interval of volcanic activity [73]. The volcanic layers at the top and at the bottom of the formation were dated radiometrically at 274.1 ± 1.6 and 276.5 ± 1.1 Ma respectively [74,75], suggesting a middle–late Kungurian age for the unit. The stratal succession within the Tregiovo Formation is composed principally of three distinct facies: (i) massive conglomerates and sandstones; (ii) laminated mudstones, siltstones and limestones; and (iii) carbonates and interbedded cherts [13,74]. The clast-supported conglomerates and coarse-grained sandstones are restricted to the base and the top of the section, whereas the thinly-bedded mudstones and fine-grained siltstones represent the central part of the succession. Quartz, pyrite, silver and lead-and-zinc mineralization was observed within these layers. Marly limestone beds with microbial structures and layers of silica (as chert) occur in the upper part of the succession. The succession was interpreted as evolving from an alluvial fan environment (Facies A) to a lacustrine environment (Facies B) and finally into an ephemeral saline lake typical of an arid environment (Facies C). The fine-grained sediments of facies B and C are rich in fossils, particularly plants, vertebrate and invertebrate sedimentological trace fossils, conchostracans and palynomorphs.

#### Tregiovo flora

Somewhat more than 450 plant fossils are preserved as impressions or strongly coalified compressions. Some specimens are covered by thin mudstone layers resembling sediment casts. Pyritization is common. The plant assemblage consists of fronds, leaves, shoot and stem fragments and reproductive organs of horsetails, pteridophytes, possible pteridosperms, coniferophytes ginkgophytes and taeniopterids (S1 and S2 Tables) [13]. Horsetails are rare in the flora and restricted to stem fragments of *Annularia*, impressions of stem fragments and internodal diaphragms marked with longitudinal vascular bundles that belong to *Calamites* [13]. Several frond fragments are associated either with ferns or pteridosperms, such as *Lodevia nicklesii*, which is represented by isolated flabelliform pinnules (20 x 40 mm) divided into 5 to 7 main lobes that preserve a distinct midvein and delicate lateral veins [13]. Another element is *Sphenopteris kukukiana*, characterized by bipinnate frond fragments with a rachis bifurcating basally and ovate pinnules attached by their blade bases to the axes [13]. Also present are umbrella-shaped, ovuliferous discs with a lobed margin that have been assigned to *Peltaspermum*.

The remaining seed plants are ginkgophytes, taeniopteroids, cordaites and conifers. Putative ginkgophytes include wedge-shaped leaves, about 35 by 50 mm in mature dimensions, with irregularly bifurcated, stripe-like segments 0.5 to 2.5 mm by 2 to 8 mm, resembling *Esterella gracilis* [13]. Taeniopteroid leaves are preserved as two types: (i) narrow, 35 mm wide, lanceolate leaves, each with a pointed apex that has delicate veins; and (ii) wider leaves displaying a convex apex, broad midrib and distinct, densely inserted veins, up to 20 per cm. The taeniopterid *Lesleya* is represented by small leaf fragments with secondary veins inserted at an acute angle along the midrib [77].

Conifers are the most diverse plant group [13,77]. *Hermitia geinitzii* is characterized by helically attached leaves that are roundish to elongate in overall shape and bear a rounded apex [13]. In *Hermitia* sp. the leaves are narrow and falcate (0.3–2.5 x 2–10 mm) and characterized by a curved apex and imbricating bases. *Hermitia* sp. specimens are plagiotropic (horizontally deployed) shoot fragments with lateral branchlets arising oppositely and within a narrow angle of 50–70°. *Walchia* leaves are narrow and falciform, bearing an acute apex and sometimes overlapping bases. The plant assemblage additionally includes big-leaved conifers. *Ullmannia* sp., for example, is represented by broad, falcate leaves (4−15 x 1.5−4.5 mm) with decurrent bases, a distinct midvein, and curved, pointed apices [77]. In *Ortiseia*, the leaves are inserted at a wide angle and occur in a loose helix on the axis. *Ortiseia* leaves are bifacial, triangular, ovate or obovate, with a rounded to obtuse apex that occasionally recurves towards the axis. *Feysia*, by contrast, is characterized by narrow, slightly falcate leaves with a decurrent basis, bearing dimensions of 0.5 to 1.2 mm by 1.5 to 5 mm and an insertion pattern forming a dense helix. For *Quadrocladus*, the leaves are 1 to 4 mm by 3 to 20 mm in dimension and bear a rounded apex, a slight constricted, perpendicular attachment to the axis. *Dolomitia cittertiae* and *Pseudovoltzia liebeana* currently are represented also by female dwarf-shoots [13]. A plant of unknown affinity is characterized by having a minimally twice-bifurcating lamina with ribbon-like segments. These segments are entire-margined, bear an obtuse apex and a wide central band-like structure that perhaps are coalesced veins of a midrib. Secondary veins are not visible. In addition, of unknown botanical affinity are cordaitalean-type leaves with distinct parallel veins and possessing a thickened, crescent-shaped attachment area and serrated blade margins [13].

### Early Lopingian (Wuchiapingian)

#### Gröden/Val Gardena Sandstone

The Gröden/Val Gardena Sandstone (Wuchiapingian) plant assemblage occurs at Bletterbach Gorge (Fig 1), which is located between the villages of Aldein (Aldino) and Radein (Redagno) (Fig 1). The Gröden/Val Gardena Sandstone unconformably overlies Cisuralian andesites, rhyolitic lavas and ignimbrites of the Athesian Volcanic Group, and is separated from the volcanic sequence by a sedimentary hiatus of approximately 14 to 27 million years [78,79]. The base of the Gröden/Val Gardena Sandstone is characterized by poorly sorted conglomerates, pebbly and muddy sandstones and siltstones [80]. The stratigraphic succession details alternations of fluvial siliciclastic, evaporite and mixed carbonate and siliciclastic deposits, reflecting environments of alluvial fans, braided rivers, shallow channels, coastal sabkhas and evaporitic lagoons on a shallow, epicontinental shelf [81,82]. Continental sabkha episodes are exemplified by sparse accumulations of nodules and continuous layers of gypsum [80]. Paleosols such as calcic soils [81] suggest a warm to hot, semi-arid to dry-subhumid climate with strong seasonality [78].

At about 80 m from the base of the succession, an increasingly marine influence can be observed by a color change from red to grey to blackish, as well as wavy bedding or inclined heterolithic stratification, typical of a tidally-influenced coastal plain [80,83,84]. A thick, marine carbonate bed with sparse cephalopods marks the maximum extent of the marine transgression [78,80]. Grey to blackish mudstones crop out above the carbonate bed, indicating a tidally-influenced environment. Cross-bedded, shallow channel deposits and immature paleosols (entisols) provide evidence for the return of continental conditions. The transition of the Gröden/Val Gardena Sandstone to the overlying, brackish to marine Bellerophon Formation is indicated by interfingering fluvial and coastal-lagoonal deposits. The Bellerophon Formation is characterized by lagoonal dolomicrites rich in evaporitic gypsum and open-lagoonal dolomitic packstones [80,85,86]. Palynological and tetrapod data suggest a Wuchiapingian age for the Gröden/Val Gardena Sandstone at Bletterbach Gorge [84].

The Gröden/Val Gardena Sandstone is noteworthy for its ichnofauna [86–91]. Tetrapod footprints from this deposit are attributable to vertebrate groups, such as Gorgonopsia, Cynodontia, Lepidosauromorpha, Pareiasauridae and Rhynchosauroidea [92]. Typically Permian ichnogenera are *Ichniotherium* and *Hyloidichnus*, found in association with taxa of clear Triassic affinity such as *Rhynchosauroides* [92,93]. Sparse plant remains were collected from several stratigraphic levels within the Gröden/Val Gardena Sandstone, but units below and above the carbonate bed of maximum transgression are very abundant and well preserved [84,94–100].

#### The Gröden/Val Gardena flora

About 1870 plant specimens from the Gröden/Val Gardena Sandstone were assigned to 30 fossil taxa, including impressions and compressions of foliage, shoots, stem fragments, fructifications, and dispersed seeds (S1 and S2 Tables). Horsetails are represented by sporophylls (4−6 mm in diameter) and leaf-sheath fragments (20−40 x 40−70 mm). Pteridosperms are represented by frond fragments and reproductive organs of *Germaropteris martinsii*. The fronds are characterized by small, coriaceous pinnules with a semicircular shape and broad attachments to the rachis. The ovuliferous organs are of the *Peltaspermum*-type [101]. *Sphenopteris suessii* is characterized by inversely lanceolate pinnules (9 x 13 mm) with a constricted base. In *Sphenopteris* sp. 1, the pinnules dichotomously fork more or less irregularly. Additional elements are small pinnae fragments with alethopteroid-type pinnules. The pinnules are oblong to slightly falcate with a rounded apex and a decurrent, proximal base; adjacent pinnules are connected by a wing along the rachis [101].

Three different types of leaves are affiliated with cycadophytes. *Taeniopteris* sp. A has narrow, elongate, entire-margined leaves with the lamina inserted on the upper part of the rachis. This species is characterized by secondary veins and 15−18 veins per centimeter that do not bifurcate. By contrast, *Taeniopteris* sp. B has leaves that are much broader, 70–130 mm wide, and there are 12−18 veins per centimeter that bifurcate once, close to the midvein. A cycad-like leaf is present and characterized by narrow, linear segments [99]. Similarly, the ginkgophytes are characterized by several, different leaf types. *Baiera digitata* is subdivided into a distinct, elongate petiole and wedge-shaped lamina. The lamina is subdivided at least once in a dichotomously branching, ginkgoalean pattern, and the segments are linear to slightly elliptical with a convex apex and eight, parallel veins in the ultimate segments [100]. However, *Sphenobaiera* sp. A lacks a petiole and has leaves that are deeply incised in a dichotomously branching, pattern. *Sphenobaiera* lobes are broadly lanceolate with convex or pointed apices. The veins are delicate and bifurcate several times. These ginkgophytes probably are affiliated with roundish to ovoidal ovules and seeds, some perhaps attached to leaves typical of *Baiera digitata* [96]. Other, enigmatic leaf fossils also may be of ginkgophyte origin. These leaves include narrow, strap-like segments which fork occasionally and resemble leaves of *Trichopitys*, *Polyspermophyllum* and *Dicranophyllum*. Another enigmatic leaf type represents a wedge-shaped lamina characterized by a distally enlarged middle segment. This leaf type somewhat resembles certain species of the enigmatic genus *Psygmophyllum*.

The conifers, as in the Cisuralian Tregiovo Flora, are the most diversified group. *Ortiseia leonardii* shoots are covered by helically arranged, sessile leaves. The leaves are broadly oval with a pointed apex [95,102]. In *Ortiseia visscheri* the leaves are smaller and inserted in a tighter helix. Another conifer, *Pseudovoltzia liebeana*, is characterized by more needle-like and helically arranged leaves. The leaves are heterophyllous, with falcate leaves bearing a pointed apex and elongate leaves possessing a broad basis [95,96,103]. *Quadrocladus* has elongate, narrow leaves (1.5−2 x 35−40 mm), with rounded apices [95,104]. *Dolomitia cittertiae* has small, helically arranged, adpressed leaves with rounded apices [95,96,104,105]. This flora also has yielded several female and male cones, charcoal remains and permineralized wood [106].

### Early Middle Triassic (Anisian)

#### Agordo, Dont and Richthofen Formations

Anisian plant assemblages originate from several localities in the Dolomites of Northern Italy (Fig 1). The most important are the Kühwiesenkopf (Monte Prà della Vacca) Flora of the Dont Formation, the Furkelpass (Passo Furcia) Flora of the Richthofen Formation near Olang (Valdaora) in the northern Dolomites, and the Valle San Lucano Flora of the Agordo Formation in the central Dolomites. The Hochalpenkopf, Mauerkopf and Monte Rite floras of the Dont Formation were exceedingly depauperate and were not included in the plant–insect analyses.

The Dont Formation is considered as a hemipelagic, carbonate-terrigenous sequence formed in a marginal basin environment [107–113], and dated as middle to late Pelsonian [114,115]. This unit is positioned on top of a massive carbonate platform of the early Anisian (Bithynian) Gracilis Formation [109,111]. Throughout the entire Dont section, variable numbers and thicknesses of lens-shaped fossiliferous layers alternate with silty and marly limestone layers. The entire succession is rich in fossils, especially bivalves, brachiopods, ammonoids, gastropods and crinoids. Most of the plant remains are restricted to a single fossiliferous horizon about 1 m thick and co-occur with marine, brackish and freshwater fishes, a terrestrial reptile and marine invertebrates [113]. This horizon was interpreted as a “…very rapid burial event caused by gravity flows within a marine basin in connection with heavy storms in the terrestrial domain” [113].

The lower part of the Dont Formation can be considered equivalent to the Voltago Conglomerate and the noted “*Voltzia* beds” of Recoaro [110]. The middle to upper part of the Dont Formation is coeval with the Agordo Formation in the central Dolomites. The Agordo Formation is dominated by bioclastic calcarenites that alternate with fine-grained sandstones and dark limestones [11,116]. Superposing the Dont Formation and the Agordo Formation is the Richthofen Conglomerate or, along its basinal extent, a marine equivalent. The Richthofen Conglomerate is dominated by red sandstones, siltstones and conglomerates. It has been interpreted as being deposited in a relatively arid, fluvial or otherwise transitional continental to marine environment [74,109,110]. The lower part of the Richthofen Conglomerate occasionally is rich in plant fossils [117], especially in lenses of grey- to buff-colored, fine-grained siltstones and in marly and carbonate siltstones that indicate a marine source. These finer-grained lenses contain bivalves, brachiopods, gastropods and crinoids.

#### The Agordo, Dont and Richthofen floras

The plant assemblage in the Agordo Formation at Valle San Lucana (Fig 1) is poorly developed and fragmentarily preserved, indicating long-distance transport of plant material to the area of deposition. The plant remains consist of horsetails, ferns, pteridosperms, cycadophytes and conifers (S1 and S2 Tables). The horsetails are represented by stem and rhizome fragments of *Equisetites*. Of the several ferns present, one prominent form is *Anomopteris mougeotii*, a bipinnate, herbaceous fern with aphlebiae at the bases of linear pinnae that contain rounded to slightly auriculate pinnules. *Neuropteridium voltzii* is another fern that has once-pinnate fronds up to 1 m long that bear falcate pinnae characterized by neuropteroid venation; its fertile fronds belong to the genus *Scolopendrites*. A third fern, *Cladophlebis remota*, is bi- to tripinnate with pinnules attached along their entire base to the rachis in a typical pecopteroid fashion. Presently, the only definitive pteridosperm in this deposit is an umbrella shaped reproductive organ of the *Peltaspermum* type. Cycadophytes are represented by small, entire-margined leaves of *Taeniopteris* sp. The most prominent conifer is *Voltzia recubariensis*, characterized by robust, triangularly and helically arranged leaves arising almost perpendicularly from the axes. Other conifer shoot fragments belong to *Albertia*.

At Kühwiesenkopf (Fig 1), more than 3000 plant remains are attributed to vegetative and reproductive organs of 37 taxa of lycophytes, horsetails, ferns, pteridosperms, cycadophytes and conifers [118]. Lycophytes are represented by both herbaceous and subarborescent taxa [118]. *Selaginellites leonardii* is present as small, heterosporous strobili and dichotomously branching sterile axes bearing small, subtriangular leaves. *Lepacyclotes bechstaedtii* has scale-like sporophylls that are basally inserted on stems while its elongate, sterile leaves are arranged along the central part of the plant. *Isoetites brandneri* is characterized by a short stem, from which arise long, lanceolate leaves provided with micro- and macrosporangia occurring at the expanded leaf bases. *Lycopia dezanchei* is a subarborescent plant with a dichotomizing, creeping rhizome and 1 to 2 m high, elevated “stems” that usually bifurcate once apically. These erect rhizomes are covered with helically arranged, long, lanceolate leaves with thick, papillate cuticles [118]. Horsetails are represented by three taxa. *Equisetites mougeotii* has a hypogenous rhizome with short internodes that give rise to vertical, unbranched stems bearing whorls of microphylls and strobili attached serially at multiple nodal levels. *Neocalamites* is a branched, arborescent horsetail with long and narrow leaves attached in whorls at the nodes [119,120]. *Echinostachys* occurs as isolated, equisetalean strobili possessing helically arranged sporophylls that appear in an approximately rhomboidal profile.

The pteridophytes at Kühwiesenkopf exhibit the highest species diversity of all plant groups within the flora [121]. *Neuropteridium elegans* and *Neuropteridium voltzii* have once-pinnate fronds with pinnae characterized by neuropteroid venation. The former has more diminutive fronds [122], and smaller, more roundish pinnules. Both species are characterized by the fertile fronds *Scolopendrites grauvogelii* and *S*. *scolopendrioides*. *Gordonopteris lorigae* is a tripinnate fern bearing relatively small, rounded pinnules with a neuropteroid venation. *Danaeopsis* sp. cf. *D*. *angustifolia* [106] is represented by lanceolate pinnae with a strong midrib and secondary veins forking once near the midrib and along the middle to outer part of the lamina; anastomoses are rare. Bipinnate fronds with pinnules are characterized by a distinct midrib and secondary veins that arise at an acute angle, curving and bifurcating along the central section of the lamina, ending perpendicularly at the margin. These forms have been assigned preliminarily to *Marattiopsis*. *Sphenopteris schoenleiniana* displays small, tripinnate fronds with a very delicate rachidial organization and falcate pinnules sometimes featuring an undulate to incised margin and veins. Other pteridophytes, known also from the slightly younger Agordo plant assemblages, are *Anomopteris mougeotii* and *Cladophlebis remota*. A second form is a bipinnate, *Cladophlebis*-type leaf that bears smaller, subtriangular pinnules with a strong midrib. An indeterminable fern frond consists of rhomboidal pinnules emerging from a stout rachis. The venation of the latter form arises from the lower, basal angle of the pinnules as an undeveloped midvein, with some lateral veins that fork several times. A fertile organ of unresolved botanical affinity–either a pteridophyte or pteridosperm–is *Lugardonia paradoxa*, and is composed of helically arranged short stalks, each of which bears a cluster of 3 or 4 elongate microsporangia.

The most abundant taxon among the pteridosperms of Kühwiesenkopf is the pinnate-leaved *Scytophyllum bergeri*. The size and shape of the pinnae of this species varies noticeably with respect to their growing position on the trunk. Sun leaves have lanceolate pinnae, with an entire margin and a decurrent lower lamina. By contrast, shade leaves bear pinnae that are broadly lanceolate with a crenate-lobate to undulate margin and a decurrent proximal and restricted distal lamina attachment [123]. Umbrella-shaped ovuliferous discs, each with minimally 15 marginal lobes that mature to seeds, are assigned to *Peltaspermum bornemannii* and belong probably to *Scytophyllum bergeri* [123]. The leaflets of *Sagenopteris* are ovoid, up to 55 mm long, and are supported by a strong midrib extending almost to the margin and by anastomosing lateral veins that form wide meshes. *Ptilozamites* sp. cf. *P*. *sandbergeri* is characterized by pinnate fronds with rectangular pinnules that have a narrow, vein-free border and are attached to the rachis along the entirety of their base.

Cycadophytes are well represented in the flora, particularly by *Bjuvia*, which is characterized by long, entire-margined leaves (20 by 50 cm in dimension) and possessing a strong midrib and distinct, parallel secondary veins [113]. At least two leaf types belong to *Taeniopteris*. The *Taeniopteris* leaves are smaller than those of *Bjuvia*, and have a thick midrib with undivided lateral veins. Pinnate leaf fragments with strap-like, regular or irregular leaf segments resemble *Nilssonia neuberi*. Female fructifications are present, both as dispersed macrosporophylls and cones that belong to *Dioonitocarpidium*. The macrosporophylls consist of a finely pinnate apical portion and a basal fertile region with two rows of 8–12 seeds.

The conifers are represented by various species of *Voltzia*, such as the typical Anisian taxon *Voltzia recubariensis*. *Voltzia walchiaeformis* has fine, falciform needles inserted loosely on the axis, the secondary shoots of which are attached in one plane to the primary shoot. *Voltzia* sp. is a heterophyllous species with leaves ranging from short, falcate, 1 cm long, and with a pointed apex, to those that are long, strap-like leaves, 3 to 4 cm long and bearing a rounded apex. In *Albertia*, the multiply branched shoots are covered by elliptic leaves attached in a loose helix. *Pelourdea vogesiaca* is characterized by up to 25–30 cm long, lanceolate leaves with crescent-shaped bases and distinct parallel veins [113].

The coeval plant assemblages of Hochalpenkopf, Maurerkopf and Monte Rite (Fig 1) are considerably less diverse as those of Kühwiesenkopf, and are poorly preserved. The plant remains were attributed to the horsetail *Equisetites mougeotii* at Maurerkopf, the pteridophyte *Gordonopteris lorigae* and the pteridosperm *Peltaspermum bornemannii* at Hochalpenkopf, and the conifer *Voltzia* sp. at Hochalpenkopf and Monte Rite.

The plant assemblage at the Furkelpass (Fig 1) is represented by compressions and impressions of horsetails, pteridophytes, pteridosperms, cycadophytes and conifers [117]. The assemblage is fragmentary and poorly preserved. Lycophytes are represented by *Lycopia dezanchei*, and horsetails by poorly preserved stem fragments of the *Equisetites mougeotii* type. Pteridophytes are more abundant, with frond fragments of *Anomopteris mougeotii*, *Gordonopteris lorigae*, *Sphenopteris schoenleiniana*, *Cladophlebis remota*, *Cladophlebis* sp. 1, *Neuropteridium elegans*, *Neuropteridium voltzii*, as well as their fertile fronds *Scolopendrites*. *Cladophlebis leuthardtii* has small, falcate pinnules 2 to 3 mm long that are attached perpendicularly on the rachis. Some umbrella-shaped, discoid structures belong to the pteridosperm *Peltaspermum bornemannii* that probably represent the female reproductive organ affiliated with the fronds of *Scytophyllum bergeri*, also preserved in this flora. An additional pteridosperm is *Sagenopteris* sp. Cycadophytes are represented by the large-leaved *Bjuvia dolomitica*, the small-leaved *Taeniopteris* sp., and *Dioonitocarpidum*-type reproductive organs. Presently, *Voltzia recubariensis* is the only conifer from this plant assemblage.

### Late Middle Triassic (Ladinian)

#### The Vulcanites, Fernazza and Wengen/La Valle Formations

Late Middle Triassic plant assemblages originate from several localities in the Dolomites (Fig 1). The most important localities are: (i) Monte Cernera near Cortina, of the Aquatona Formation; (ii) Monte Agnello near Predazzo, occurring in the Vulcanites; (iii) Forcella da Cians/Ritberg near Wengen/La Valle; (iv) Seewald, the latter two are part of the Fernazza Formation; and (v) Innerkohlbach, near Prags/Braies, belonging to the Wengen/La Valle Formation. During the Ladinian Stage, the Dolomites were subject to significant volcanic activity. Previous to vulcanism, successions of laminated beds of carbonates, siltstones, marls and sandstones occur, but also distinctive “pietra verde” strata and ialoclastic tuffs that often occurred as turbidites in basinal deposits. The latter features are related to the commencement of volcanic activity.

The volcanic complexes were mostly submarine, although locally, such as in the Predazzo area, subaerial eruptive centers also existed [124,125]. At Monte Agnello the volcanic succession overlies the Sciliar Dolomite, a carbonate platform of late Anisian to Ladinian age. This volcanic succession is composed of “explosion breccia” at the base, succeeded by lava breccia and alternations of lava flows and tuffs [126–128]. The greyish-greenish clastic rocks of the “explosion breccia” [126] are represented by calcareous, volcanic and metamorphic rock fragments, clastic rocks and isolated crystals that are bound by carbonate and/or chlorite-serpentine cement [126]. The succession accumulated in an essentially subaerial environment that is attributable to falls and surges deposited as sandwaves occurring in a massive facies consisting of volcanic bombs, antidunes and accretionary lapilli. The plant remains of Monte Agnello were preserved in the tuffs below the “explosion breccia”. These tuffs are coarse to fine-grained in lithology and locally rich in accretionary lapilli, or more rarely, small lithic lapilli.

The Fernazza Formation is a volcanic–turbiditic succession containing tuffites, ialoclastites, pillow lavas and pillow breccias. During the interruptions of volcanic episodes, debris flows and submarine avalanches occurred [129]. The succession was deposited in basinal settings with marked lateral differences. Based on its ammonoid fossils, these strata have been dated to the late Longobardian, equivalent to latest Middle Triassic [129–132]. By contrast, the Wengen/La Valle Formation is characterized by terrigenous-carbonatic successions emplaced at the top of volcanoarenites of the Fernazza Formation, and is covered by the St. Kassian/San Cassiano Formation. This latter unit was dated by ammonoid biostratigraphy as latest Longobardian.

#### The Vulcanites, Fernazza and Wengen/La Valle floras

The plant assemblage at Cernera (Fig 1), of the Aquatona Formation, is composed principally of pteridophyte frond impressions and compressions, cycadophyte leaves and conifer shoots (S1 and S2 Tables). The pteridophytes are represented by *Anomopteris mougeotii*, *Gordonopteris lorigae*, *Cladophlebis leuthardtii*, *Neuropteridium* sp. and *Marattiopsis* sp. The cycadophytes are composed of big-leaved taxa with an entire margin, such as *Bjuvia dolomitica*, but also segmented leaves like *Sphenozamites wengensis* and "*Pterophyllum"* sp. Among the conifers, taxa with falcate-shaped leaves such as *Voltzia dolomitica* are associated with broad-leaved forms that include *Albertia* sp. and *Elatocladus*. The latter conifer is characterized by helically attached, elongate leaves with a rounded apex and a slightly reduced basis area.

The plant assemblages at Monte Agnello (Fig 1) occur within the Vulcanites and are preserved as impressions or almost three-dimensional casts accompanied by completely degraded organic material. In some cases the foliage of especially large-leaved plants are characterized by perforations related to volcanic lapilli present in the rock. Horsetails are few and poorly preserved, some of which are present as fragments, attributable to *Schizoneura paradoxa*. The ferns are represented by Osmundaceae (*Neuropteridium elegans*), Matoniaceae (*Phlebopteris fiemmensis*) and presumptive Dipteridaceae (*Thaumatopteris* sp.). *Phlebopteris fiemmensis* is characterized by up to fourteen pinna fragments arising from two short rachidial arms. The overall lanceolate-shaped pinnae are composed of lanceolate to falcate pinnules that display broadly attached bases and pointed apices. The midvein is slightly undulating in the apical part of larger pinnules; lateral veins are delicate, simple or once bifurcated near the base. The leaf fragments of *Thaumatopteris* sp. have distinctly reticulate, coarse meshes that become more finely partitioned at the margins. *Chiropteris monteagnellii* has funnel-shaped leaves that bear an elongate, thick petiole and spatulate, secondarily incised laminae. The anastomosing venation forms very narrow meshes that lack a “midrib”. The bipinnate fronds of *Cladophlebis ladinica* have a stout, winged rachis, with basiscopically enlarged rhomboidal to falcate pinnules and fused basal pinnae. Pinnular midribs are distinct, with lateral veins bifurcating up to three times. *Cladophlebis* sp. has small pinnules (2.5‒4 x 5‒11 mm), is lanceolate to slightly falcate in shape with rounded apices, and has a broad base with secondary veins that arise at an acute angle. The pteridosperms occur as *Scytophyllum bergeri* and the cycadophytes are present as the entire-leaved genera *Bjuvia* and *Taeniopteris*, but also by taxa with segmented leaves such as *Nilssonia neuberi* and *Apoldia* sp. Conifers are represented by shoots of *Voltzia* sp. and *Pelourdea vogesiaca*.

The plant assemblage occurring at Forcela da Cians/Ritberg (Fig 1), of the Fernazza Formation, yielded horsetails belonging to *Equisetites arenaceus*, frond fragments of the pteridophytes *Cladophlebis leuthardtii*, *Gordonopteris lorigae* and *Neuropteridium* sp., and the pteridosperm *Ptilozamites sandbergeri*. The cycadophyte *Bjuvia dolomitica* is characterized by large, more than 60 cm long, entire-margined leaves with secondary veins that emerge perpendicularly from the rachis and reach the margin with a concentration of 14‒18 veins per cm. The leaf segments of *Sphenozamites wengensis* are small, spatulate and attached oppositely or suboppositely to the upper side of the broad rachis. Additional, small leaf fragments belong to *Taeniopteris* sp. The conifers are the most diversified group in the flora, and include with *Pelourdea vogesiaca* and several *Voltzia* species. *Voltzia dolomitica* has penultimate and ultimate shoots of the same width, the latter arising at an angle of 45°. The leaves of this species are helically attached, falcate to triangular in shape, with a more or less acute apex, and partly overlap onto the shoot. *Voltzia pragsensis* has ultimate shoots arising at an acute angle, 30‒45˚, and are densely spaced from the penultimate shoot. Leaves of *V*. *pragsensis* are densely appressed to their axes, and triangular in shape with an acute apex. *Voltzia ladinica* has heterophyllous leaves that are falcate to elongate in shape typically 1‒2 x 4‒10 mm, but to up to 25 mm in length, and with ultimate shoots arising at angles of approximately 60°.

The plant assemblage found at Seewald near Prags/Braies (Fig 1), is part of the Fernazza Formation, and is composed of a variety of seed-plant taxa. These include pteridosperm frond fragments (*Ptilozamites sandbergeri*), cycadophyte leaf fragments such as “*Pterophyllum*” sp., *Sphenozamites wengensis* and *Taeniopteris* sp., as well as conifer shoots and cones belonging to *Pelourdea vogesiaca*, *Voltzia dolomitica*, *V*. *ladinica* and *V*. *pragsensis*. “*Pterophyllum*” sp. is characterized by highly divided leaves, with each leaf segment exhibiting an elongate to rectangular shape with a parallel venation [15,133,134].

The plant assemblage of Innerkohlbach, also near Prags/Braies (Fig 1), occurs in the Wengen/La Valle Formation. This assemblage includes pteridophyte frond fragments such as *Cladophlebis leuthardtii*, *Danaeopsis angustifolia* and the pteridosperm *Ptilozamites sandbergeri*. Also present are shoot fragments and cones belonging to the conifers *Pelourdea vogesiaca*, *Voltzia dolomitica*, *V*. *ladinica* and *Voltzia pragsensis* [15,134].

## Materials and Methods

The procedure for conducting this study involved collection and processing of specimens, identification of the plant hosts, establishing the presence of herbivory from detritivory, allocation of herbivory to functional feeding groups (FFGs) and their damage types (DTs), and quantitative analyses. The analyses included calculation of a variety of frequency and richness measures for determining levels of total, generalized and specialized herbivory in specific plant taxa, plant groups, and floras from individual and pooled localities.

### Specimens, definitions and repositories

A quantitative analysis of herbivory was made by examination of the frequency of attack, if any, of each foliage element in the ten floras of Permian and Triassic age from the Dolomites Region. Plant–insect interactional data were based on 4242 plant specimens representing 1995 Permian and 2247 Triassic specimens that were allocated to 86 fossil species, of which 32 were Permian and 56 were Triassic in age (two taxa were boundary crossers). The plant groups examined were lycophytes, sphenophytes, pteridophytes, pteridosperms, cycadophytes, ginkgophytes, coniferophytes (conifers) and plants of uncertain taxonomic position (S1 Table). The frequency-of-attack measurements were made by reference to explicitly defined damage types, using the third version of the “Guide to Insect (and other) Damage Types on Compressed Plant Fossils” [135], and subsequent addenda, for identification of distinctive damage types (DTs) on the Dolomites Permian and Triassic floras. A foliar element was defined as any chlorophyllous, planated plant organ with a measurable surface greater than 0.5 cm^2^, and would include organs such as true leaves, pinnules, scale leaves, sporophylls, sphenopsid stems and phyllodes [17].

The examined specimens are housed in four institutions. Of the leaves examined, more than 340 slabs of Kungurian age are stored at the MUSE, Museo delle Scienze of Trento, Italy. The “MUSE PAG” prefix is used for specimens from the Tregiovo locality that are part of the “Ferruccio Valentini Collection”. About 500 slabs are from Wuchiapingian strata of the Gröden/Val Gardena Sandstone; about 1200 slabs are from the Anisian; and about 150 slabs are from the Ladinian of the Dolomites, all of which are stored at the Museum of Nature South Tyrol in Bozen/Bolzano, Italy, where they are curated under the “PAL” prefix. Other tens of specimens, including the Wuchiapingian Gröden/Val Gardena Sandstone, are stored at the Laboratory of Palaeobotany and Palynology at Utrecht University, The Netherlands, and are curated under the “BUT” prefix. Approximately 1000 specimens from the Monte Agnello Flora are stored at the Museo della Geologia di Predazzo, and are housed under the “MGP” prefix. No permits were required for the described study, and all relevant regulations were complied with.

### Differentiating herbivory from detritivory

Four explicit criteria were used to establish herbivory (consumption of live plant tissues) on the examined plants, rather than referring insect damage to the default category of detritivory (consumption of dead tissues) [136,137]. The first criterion consists of the presence of callus or other reaction tissue typically expressed as upraised rims of dark, botryoidal matter along cut leaf margins [138]. A second type of evidence is the occurrence of distinctive micromorphological features of interactions that would reveal the presence of mouthpart activity. Examples would include evidence for stylet sheaths by a surrounding crater of reaction tissue, common in modern piercing and sucking [139], or successive deployment of cuspule-like chew marks that would indicate chewing activity by a mandibulate insect [140]. Third, there is the presence of recurrent stereotypy in the patterns of damage, both in the structure of each individual unit of damage, such as many galls [141], and in the pattern of their collective occurrence on a plant-organ surface [142]. And last is the preferential targeting of particular plant species, organs or tissues, indicating herbivory [143], rather than the typical nonselective consumption of plants seen in detritivory [144] or attributable to physical damage [145]. These four features, collectively, could resolve almost all instances of herbivory. In very few cases there was uncertainty as to whether an interaction could be ascribed to herbivory. Accordingly, such instances were allocated to the default category of detritivory and not included in subsequent analyses. A fifth potential criterion, taxonomic uniformitarianism, employed in other studies [135,137], was not used, as none of the more modern, lower ranked insect-herbivore groups, nor their plant-host lineages were present during the examined P-Tr interval.

### Functional feeding groups and damage types

The insect functional feeding group (FFG) approach and subordinate damage type (DT) system for evaluating insect damage on fossil foliage was used to evaluate Permian and Triassic herbivory. Procedurally, an initial determination was made if instances of insect damage from various examined floras from the Dolomites Region constituted herbivory, using the four explicit criteria mentioned above. Then, the spectrum of insect damage was categorized into the FFGs collectively represented in the datasets [135]. The seven FFGs are (i) external foliage feeding, with its four subordinate groups of hole feeding, margin feeding, skeletonization and surface feeding; (ii) piercing and sucking; (iii) oviposition; (iv) galling; (v) leaf mining; (vi) seed predation; and (vii) wood boring. While oviposition is not a feeding strategy, it does have an extensive fossil record [68], has been treated as a FFG in other analyses of insect herbivory in the fossil record [17,23], and was used as such in this analysis. Fungal and other pathogen related damage, often resulting from secondary infection and alteration of plant tissues related to insect herbivory [32], was assigned to DT58 as generalized fungal infection, but was not included in our analyses of herbivory.

Details of the seven functional feeding groups present at the P-Tr interval in the Dolomites Region of northeastern Italy are the following. First, *external foliage feeding*, consisted of exophytic consumption of live plant tissues, subdivided into skeletonization and margin-, hole- and surface feeding; this was the most commonly encountered ensemble of Permian damage types [33,37,39,40]. Second, *piercing and sucking* is an exophytic interaction in which an herbivore targets and feeds on fluid tissues such as phloem, mesophyll or epidermal cell protoplasts; examples were uncommon [17]. Third, *oviposition*, though not a feeding interaction, occurs when a female insect uses a piercing, ovipositor often endowed with a serrated edge at the tip of her abdomen, for inserting eggs into plant tissues; examples are numerous [11,31]. Fourth, *galling* is the most biologically complex of all major interactions, and represents arthropod-induced, abnormal cell proliferation that can occur on or in all major plant organs; examples were common [17,20,31,36,45]. In galls, the number and often size of cells are increased relative to the normal leaf condition, attributable to secretion of chemicals by the galler that disrupts the developmental growth of the plant host [141,142]. The gall-causing organism uses this structure as both a shelter and a source of nutrition for developing immatures. Fifth, *leaf mining* is an interaction in which the larva of a holometabolous insect consumes the inner tissues of leaf or other foliage, typically tissues such as mesophyll, hypodermis or foliar vasculature. [9,71,146]. Leaf mining was very rare in the Triassic [69,71,137]. Sixth, *seed predation* is comprised of a combination of diverse damage types from multiple functional feeding groups that have the common, singular feature of killing an embryonic plant within a seed or damaging an ovule such that it becomes nonviable; examples were rare [17,49,147]. Seventh, *wood boring*, is the tunneling through cambia, wood or similar indurated tissue within a live plant, but without noticeable production of response tissue; evidence for wood boring comes in two forms: smaller oribatid mite and larger insect borings [46,148,149]. Although *fungal infection* was noted in this study as an undifferentiated DT58, currently there is no provision for categorization into more discrete, diagnosable DTs [135]. Fungal damage was evident at Bletterbach, undoubtedly caused by mostly by fungal or fungal-like pathogens, although environmental conditions such as nutrient deficiencies or water stress may cause similar signs on plant hosts, such as unspecified blotches or necroses [32].

### Quantitative analyses

The procedure for quantitative assessment of herbivory was calculation of the proportion of foliar elements that had herbivory, given as a percentage of the total number of elements examined [150]. The percentage of herbivory was determined for a bulk floral basis [136], in which a value was provided for each flora or a pooled combination of Triassic floras from particular time intervals, the Anisian or Ladinian, or as a per-taxon basis [17]. In the latter case, an assessment of herbivory was made for the members of the major plant-host groups represented, such as cycadophytes or conifers. A comparative assessment was made for the insect-herbivore component community of one representative plant host from the Wuchiapingian and another plant host from the pooled Anisian floras, following a recent example from the fossil plant–insect interaction literature [17].

## Results

**Fig 2:**
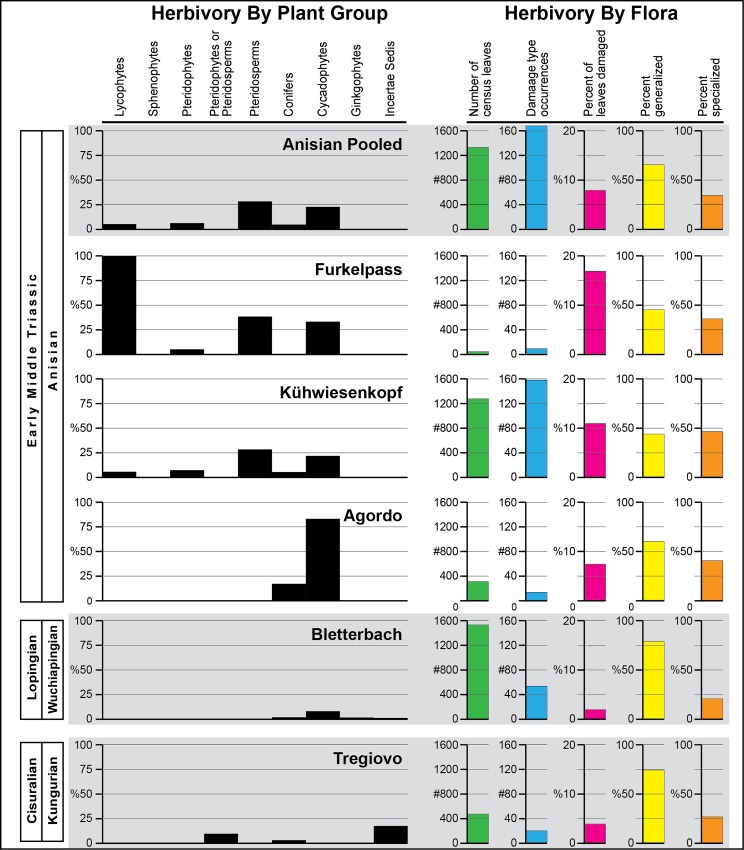
Quantitative distribution of plant–insect interactions from Cisuralian to Anisian strata in northeastern Italy.

**Fig 3:**
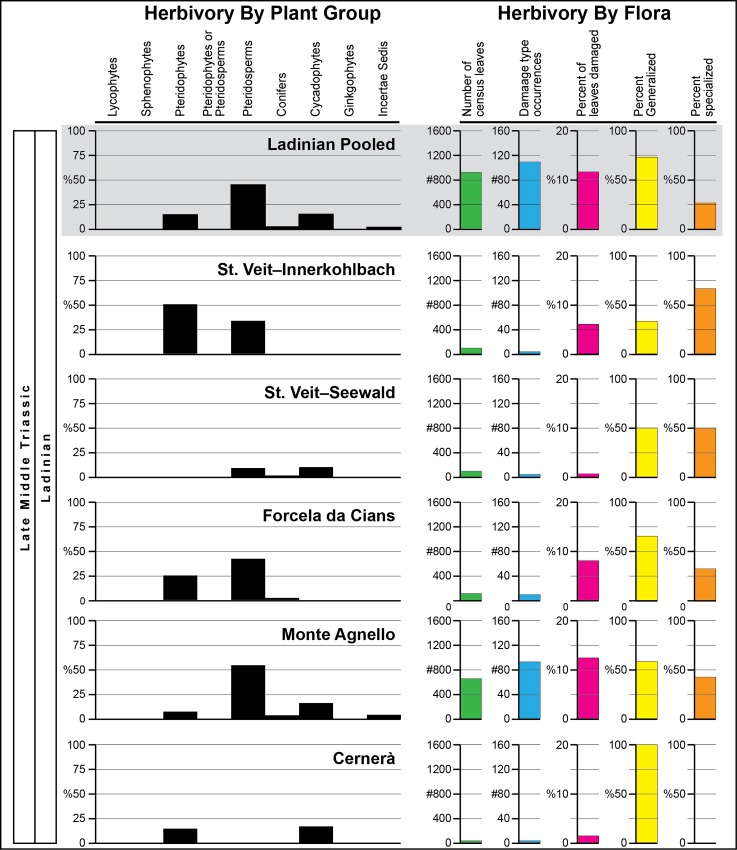
Quantitative distribution of plant–insect interactions Ladinian strata in northeastern Italy.

There are five categories of results from our study regarding the patterns of the insect herbivory in the Permian to Triassic interval from the Dolomites Region of northeastern Italy. First is a description of the flora present (S1 Table). Second is a qualitative description of the insect herbivore damage from each flora or time-specific assemblage of floras illustrated in many of the figures that follow. Third is presentation of the quantitative analysis of herbivory based on major plant groups and bulk floras from particular localities (Figs 2 and 3; Table 1; S1–S12 Tables). A fourth assessment involved establishment of plant–host generalization-to-specialization patterns (Figs 2 and 3; Table 1; S2 Table). Lastly, a comparison was made of the herbivore component–community structure between a typical plant host from the Wuchiapingian Bletterbach Flora and a counterpart from Anisian Furkelpass Flora.

**Table 1:** Damage type (DT) diversity and generalization–specialization proportions for the four major floras examined.

	Total	Generalized DTs	Specialized DTs		
Time interval	DTs	totals	percent	totals	percent
**Ladinian**					
(five pooled floras)	24	13	54.2	11	45.8
**Anisian**					
(three pooled floras)	38	15	39.5	23	60.5
**Wuchiapingian**					
(Bletterbach Flora)	16	5	31.3	11	68.7
**Kungurian**					
(Tregiovo Flora)	7	2	28.6	5	71.4

### Qualitative assessment of the insect damage

**Fig 4:**
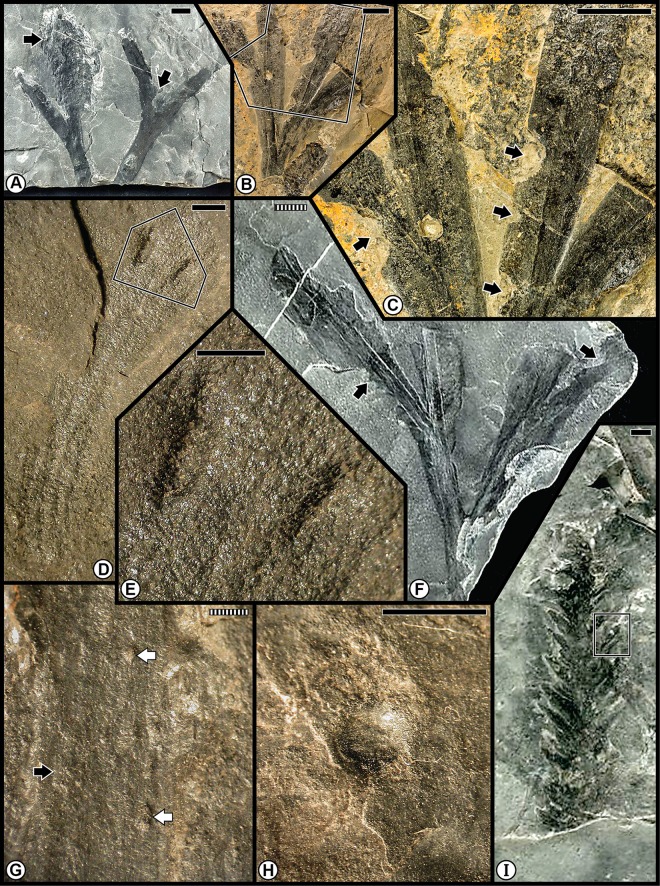
Plant–insect interactions from the Cisuralian (Kungurian) Tregiovo Flora, of the Tregiovo Formation near Tregiovo in northeastern Italy. **A**: Undetermined pteridosperm, with margin feeding (DT12) indicated by the white arrow at right, and a large stem gall (DT87) by the black arrow at left. Specimen MUSE PAG-7352. **B**: Undetermined pteridosperm with margin feeding (DT12). Specimen MUSE PAG-7094. **C**: Enlarged specimen from template at (B) showing several examples of margin feeding (DT12), four of which are indicated by black arrows. **D**: Unknown axis with two examples of stem oviposition (DT76). Specimen MUSE PAG-7176. **E**: Enlarged oviposition-mark scars indicated by template at (D). **F**: Undetermined pteridosperm showing examples of margin feeding (DT12), two of which are indicated by black arrows. Specimen MUSE PAG-7383. **G**: Foliage of the conifer *Quadrocladus* sp., showing examples of DT12 at left (black arrow) and piercing-and-sucking damage of DT46 and DT47 at right (white arrows). Specimen MUSE PAG-7116. **I**: Undetermined conifer with foliage gall (DT32), indicated by square template, enlarged in H. Specimen MUSE PAG-7204. All specimens are reposited in the Museo delle Scienze of Trento, Italy. Scale bars: striped = 1 mm; solid = 10 mm.

The Kungurian Tregiovo Flora was a species-poor-flora from the Tregiovo Formation, dominated principally by nine organ-genera of coniferophytes, but also included a few representatives each of sphenophytes, pteridophytes or pteridosperms, cycadophytes, a ginkgophyte and three taxa of unknown affinities (Table 1; S1 and S3 Tables). Almost all of the insect-mediated damage (Fig 4) has been recorded on an unaffiliated pteridosperm, probably representing a sole species that was especially prone to consumption from margin feeders (DT12), shown on several specimens (Fig 4A–4C and 4F), as well as attack from stem-related oviposition of DT76 (Fig 4D and 4E). A lesser consumed host was the coniferophyte *Quadrocladus* sp., which supported piercing and sucking damage (Fig 4G) and a gall with an outer wall surrounded by altered tissue (Fig 4H and 4I).

**Fig 5:**
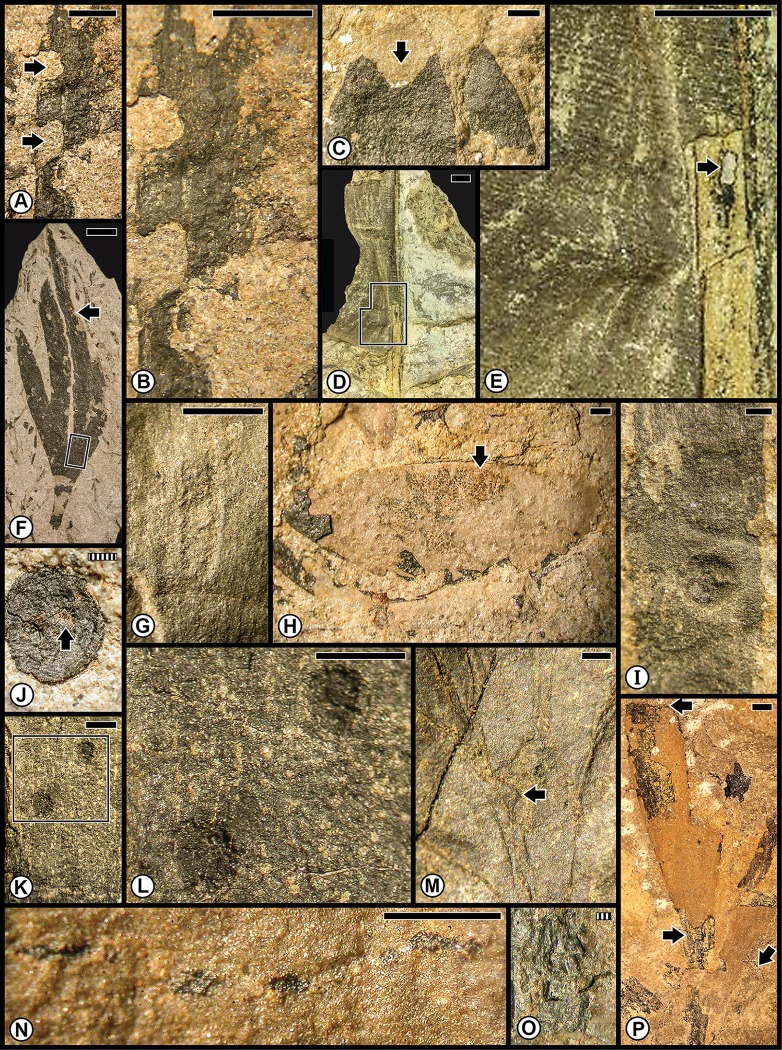
Plant–insect interactions from the Lopingian (Wuchiapingian) Gröden/Val Gardena Flora, of the Gröden/Val Gardena Sandstone at Bletterbach Gorge in the western Dolomites. **A**: Two examples at left of margin feeding (DT12), indicated by black arrows, on a degraded pinnule of the unaffiliated plant *Dicranophyllum* sp. Specimen PAL-997. **B**: Enlargement of (A), showing margin reaction tissue. **C**: Margin feeding (DT13) on the pinnular tip of the conifer *Ortiseia leonardii*. Specimen PAL-2020. **D**: An undetermined cycadophyte, showing an oviposition mark (DT76) on rachis, encompassed by polygonal template at left. Specimen PAL-1532. **E**: Enlarged image of oviposition mark in (D). The rachial epidermal apparently is absent. **F**: the ginkgophyte *Sphenobaiera nutzmani* exhibiting margin feeding (DT12) at top (black arrow) and surface feeding (DT130) below in the rectangular template. Specimen PAL-445. **G**: A magnified version of DT130 at (F), showing consumption of surface tissues. **H**: The pteridophyte *Sphenopteris* sp. 1 pinnule showing surface feeding damage (DT30), indicated by the roughened surface. PAL-1455. **I**: A globose gall (DT153) on the stem of the conifer *Quadrocladus* sp., indicated by central bulbous tissue and radiating files of cells. Specimen PAL-1464. **J**: Indeterminate, discoidal, platysperm seed with seed predation (DT74) indicated by arrow. Specimen PAL-1088. **K**: Wood borings (DT160) on an indeterminate, debarked axis. Specimen PAL-2016. **L**: Enlargement of borings at (K), showing circular cross section and infill pattern. **M**: Foliar fascicle of the conifer *Pseudovoltzia liebeana* with one foliage element showing a bulging gall (DT33) at black arrow. Specimen PAL-859. **N**: A stem of the conifer *Quadrocladus* sp., showing eight, lenticular-shaped oviposition marks (DT175) perpendicular to the stem axis. Specimen PAL-1448. **O**: An oviposition mark (DT76) on an unknown woody axis. Specimen PAL-837. **P**: The ginkgophyte *Sphenobaiera* sp., showing two major areas of surface feeding (DT30) on the blade and petiole, indicated by black arrows and margin feeding (DT12) at white arrow. Specimen PAL-821. All specimens are reposited in the Museum of Nature South Tyrol (MNS), in Bolzano, Italy. Scale bars: striped = 0.1 mm; solid = 10 mm.

The Wuchiapingian Bletterbach Gorge Flora from the Gröden/Val Gardena Sandstone is a comparatively nonspeciose flora that consists of several taxa each of pteridosperms, ginkgophytes, cycadophytes and coniferophytes (Table 1; S1 Table). The moderately diverse spectrum of insect damage in this flora is dominated by external foliage feeding, and subordinately by oviposition, galling, seed predation and wood boring (Fig 5; S4 Table), and is richer than the spectrum of herbivory on the earlier Tregiovo Flora. Bletterbach margin feeding such as DT12 has been recorded on the unaffiliated plant *Dicranophyllum* sp. and the ginkgophytes *Sphenobaiera* sp. (Fig 5A, 5B, 5F and 5P), and DT13 occurs on the coniferophyte *Ortiseia leonardii* (Fig 5C). Surface feeding, as DT30, is found on the pteridosperm *Sphenopteris* sp. 1 as well as on *Sphenobaiera* sp. (Fig 5H and 5P) and DT130 on *Sphenobaiera* sp. (Fig 5G). A single oviposition mark (DT76) is documented on the rachial tissue of an undetermined cycadophyte, where it displays inner disturbed tissue and a surrounding reaction scar (Fig 5D and 5E), and on an unidentified axis (Fig 5O). By contrast, a string of successive, end-to-end oviposition marks (DT175) formed on the stem of the coniferophyte *Quadrocladus* sp. (Fig 5N). A stem gall occurs as a globose swelling (DT153), also on *Quadrocladus* sp. (Fig 5I), and a second gall type (DT33) is present on a leaf of the coniferophyte *Pseudovoltzia liebeana* (Fig 5M). Seed predation (DT74), a very rare interaction, occurs on an undetermined platysperm seed (Fig 5J); and equally rare wood borings (DT160) are found on an unaffiliated axis (Fig 5K and 5L). This site has the broadest spectrum of functional feeding groups of any Dolomites flora, although the range of interactions is minimal on any particular host plant.

**Fig 6:**
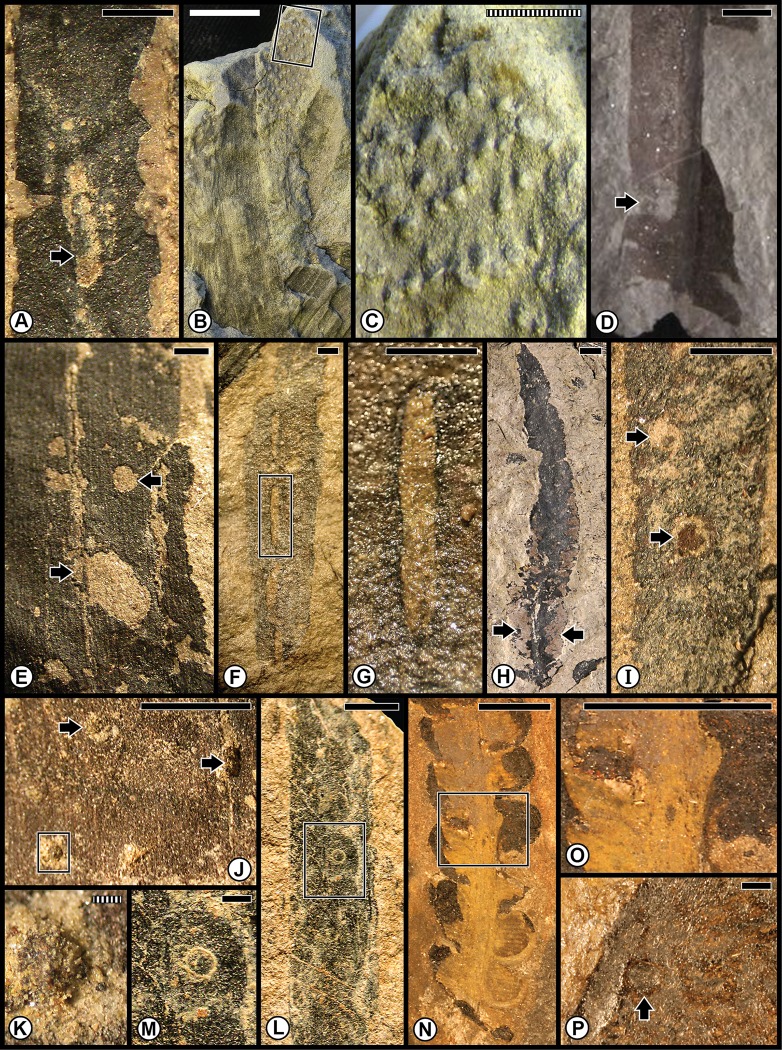
Plant–insect interactions from the Middle Triassic (Anisian) Kühwiesenkopf/Monte Prà della Vacca Flora, of the Dont Formation at Kühwiesenkopf/Monte Prà della Vacca in the northeastern Dolomites. **A**: The lycopod *Lycopia dezancheri*, showing an example of slot feeding (DT08). Specimen PAL-7603. **B**: A stem of the sphenopsid *Neocalamites asperrimus*, with a cluster of oviposition scars (DT159). Specimen PAL-11. **C**: Enlargement of a series of linear rows of oviposition scars in (B). **D**: Incised margin feeding (DT15) on an isolated pinnule of the cycadophyte *Bjuvia* sp., indicated by black arrow. Specimen PAL-1599. **E**: A leaf of the cycadophyte *Bjuvia* sp. with examples of hole feeding (DT01, DT02), indicated by arrows. Specimen PAL-294. **F**: The cycadophyte *Bjuvia dolomitica*, showing four examples of slot feeding (DT08). Specimen PAL-2147. **G**: Enlargement of slot feeding (DT08) indicated by rectangular template in (F), showing underlying matrix. **H**: Surface feeding (DT82) on both sides on the basal portion on a cycadophyte leaf of *Bjuvia* sp. Specimen PAL-729. **I**: Two miniscule galls (DT80), indicated by black arrows on the cycadophyte *Bjuvia* sp. Specimen PAL-655. **J**: Successive pinnules of the cycadophyte *Bjuvia* sp., showing several small galls (DT80). **K**: Enlargement of gall at the lower-left template in (J). **L**: The broadleaved conifer *Voltzia* sp., displaying a scale mark (DT77), circumscribed by rectangular template. Specimen PAL-1108. **M**: Enlargement of scale mark at (L). **N**: An undetermined cycadophyte, with an oviposition scar (DT76) along a rachis. Specimen PAL-11. **O**: Detail of the oviposition scar, at the juncture of the blade and rachis. **P**: The cycadophyte *Ptilozamites heeri*, with a DT32 gall. All specimens are reposited in the Museum of Nature South Tyrol (MNS), in Bolzano, Italy. Scale bars: striped = 1 mm; solid = 10 mm.

The first of the combined Anisian floras consists of the Kühwiesenkopf and related floras from the Dont Formation that collectively constitute a rather speciose assemblage. Species diversity is particularly elevated for the Kühwiesenkopf locality, the richest flora examined from the Dolomites, which contains 37 plant-organ taxa. This flora consists of lycophytes, sphenophytes, pteridophytes, pteridosperms, cycadophytes and coniferophytes, but lacks ginkgophytes (Table 1; S1 Table). The insect herbivory ranges from external foliage feeding (hole, margin and surface feeding subtypes), piercing and sucking, oviposition and galling (Fig 6; S5 Table). Small perforations of hole feeding (DT01, DT02) occur on *Bjuvia* sp. (Fig 6E), which contrasts with another type of hole feeding, slot feeding (DT08), that is present as elongate, parallel-sided, feeding holes between veins of the lycopod *Lycopia dezancheri* (Fig 6A) and on the cycadophyte *Bjuvia dolomitica* (Fig 6F and 6G). Margin feeding (DT15) is expressed as trenched incisions along the leaf margin of *Bjuvia* sp. (Fig 6D). A third type of external foliage feeding, surface feeding, is displayed along swaths on each side of a midvein (DT82) on *Bjuvia* sp. (Fig 6H). Piercing and sucking occurs as ovoidal scale marks with a central boss and associated concentric rings (DT77) on the coniferophyte *Voltzia* sp. (Fig 6L and 6M). Oviposition is emplaced as a cluster of deep, lenticular lesions with prominent lateral scars (DT159) on the stem of the sphenopsid *Neocalamites asperrimus* (Fig 6B and 6C), suggesting odonatan placement of the eggs. Oviposition also occurs as single, lenticular, insertion scars (DT76) on the massive rachis of an undetermined cycadophyte (Fig 6N and 6O). Two types of galls are present, including small hemispheroidal structures (DT80) on *Bjuvia* showing their effect on the surface of adjacent host tissues (Fig 6I–6K), and larger, walled features (DT32) on the cycadophyte *Ptilozamites sandbergeri* (Fig 6O and 6P). The broad range of interactions in the Kühwiesenkopf Flora matches the elevated number of DT occurrences.

**Fig 7:**
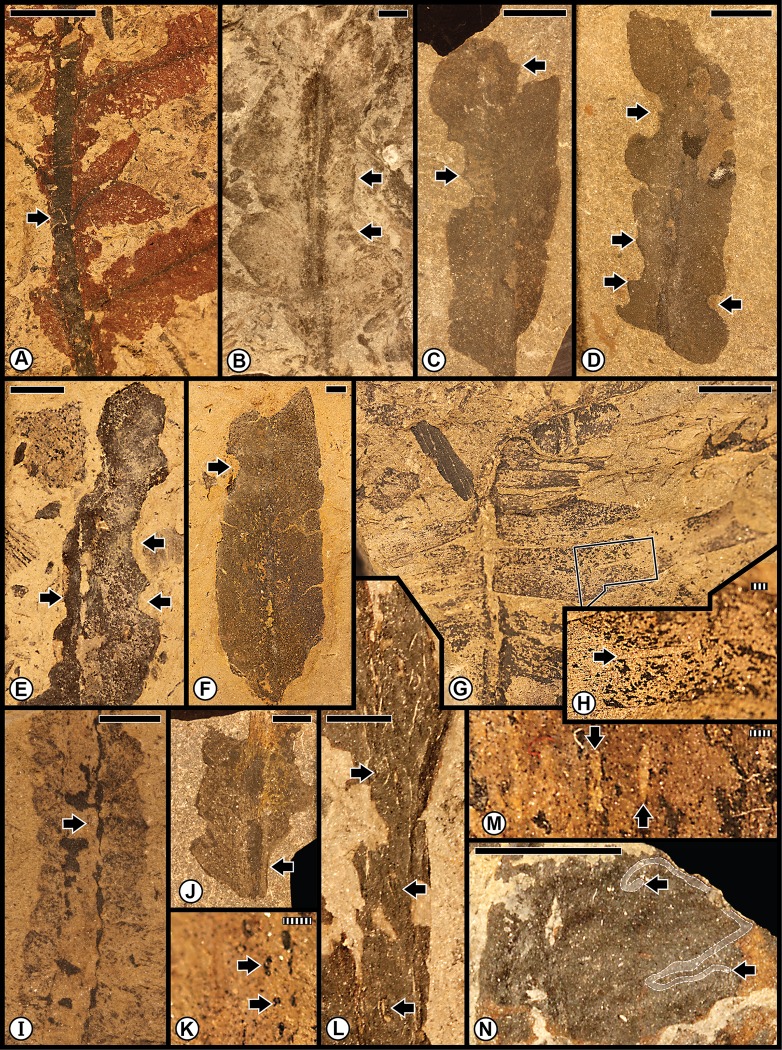
Plant–insect interactions from the Middle Triassic (Anisian) Furkelpass and related floras, of the Richthofen Formation near Olang/Valdaora in the northern Dolomites. **A**: Extensive margin feeding (DT12, DT14) of pinnular foliage on the pteridosperm *Scytophyllum bergeri*, indicated by black arrows. Specimen PAL-492. **B**: The pinnular margin of *Scytophyllum bergeri* is altered along its blade by margin feeding (DT12), indicated by black arrows. Specimen PAL-523. **C**: Another pinnule of *Scytophyllum bergeri* displaying margin feeding damage (DT12, DT143). Specimen PAL-466. **D**: Pinnule of *Scytophyllum bergeri*, exhibiting single (DT12) and multiple (DT143) examples of margin feeding. Specimen PAL-513. **E**: A *Scytophyllum bergeri* pinnule displaying single and continuous margin feeding (DT12, DT143). Specimen PAL-481. **F**: Margin feeding (DT12) on the pteridosperm *Scytophyllum bergeri*. Specimen PAL-473. **G**: A frond portion of *Scytophyllum bergeri* with several pinnules, one of which displays an example of slot feeding (DT08). Specimen PAL-469. **H**: Enlargement of pinnule in the polygon at the lower-left of (G), showing slot feeding. **I**: A specimen of showing a miniscule example of oviposition (DT76) on woody tissue, indicated by a black arrow. Specimen PAL-468. **J**: A pinnular fragment of *Scytophyllum bergeri* with extensive margin feeding (DT12, DT14) one of which is illustrated at the white arrow. Specimen PAL-483. **K**: Paired piercing-and-sucking marks (DT46, DT47) indicated by black arrows, on *Scytophyllum bergeri*. Specimen PAL-491. **L**: Ovipositional damage (DT175) to *Scytophyllum bergeri*, indicated by black arrows. Specimen PAL-510. **M**: Several examples of slot feeding (DT08), indicated by arrows, on *Scytophyllum bergeri*. Specimen PAL-518. **N**: A probable leaf mine (DT41) on a pinnule of *Scytophyllum bergeri*. Leaf mine margins are indicated by a drawn, dotted line. Main mine body indicated by white arrow and possible terminus by black arrow. Specimen PAL-517. All specimens are reposited in the Museum of Nature South Tyrol (MNS), in Bolzano, Italy. Scale bars: striped = 1 mm; solid = 10 mm.

The second group of the pooled Anisian Flora encompasses the Furkelpass/Passo Furcia and related floras from the Richthofen Formation. These floras constitute a moderately diverse assemblage of lycophytes, sphenophytes, pteridophytes, pteridosperms, cycadophytes and coniferophytes, but lack ginkgophytes and taxa of uncertain affiliation (Table 1; S1 Table). In particular, the Furkelpass Flora consists of 20 plant-organ taxa, about half of the taxa of which is present also in the Kühwiesenkopf Flora. Insect-mediated damage (Fig 7; S6 Table) consists of extensive external foliage feeding almost exclusively on the pteridosperm *Scytophyllum bergeri*. Examples of often-extensive cuspate margin feeding occur singly or continuously along the leaf-margin or extend to the midrib (DT12, DT14, DT143), and can be found on several *S*. *bergeri* pinnules (Fig 7A–7F and 7J). *Scytophyllum bergeri* also contains elongate slot feeding (DT08) paralleling the secondary venation (Fig 7G, 7H and 7M), piercing-and-sucking (DT46, DT47) occurring along the leaf margin (Fig 7K), oviposition (DT76) along the midrib of immature foliage (Fig 7I), and consecutive, lenticular-shaped oviposition (DT175) on the more sclerified axis of a mature stem (Fig 7L). Notably, *S*. *bergeri* houses a probable serpentine leaf mine with sporadically occurring frass (DT41) coursing along the margin and inner blade region (Fig 7N). This documentation illustrates the variety of DTs from several functional feeding groups that is present on the foliage of *S*. *bergeri*. Interactions on other hosts include excisions on the leaf apex and margin (DT12, DT12) on the foliage of the cycadophytes *Bjuvia* sp. and *Taeniopteris* sp.

**Fig 8:**
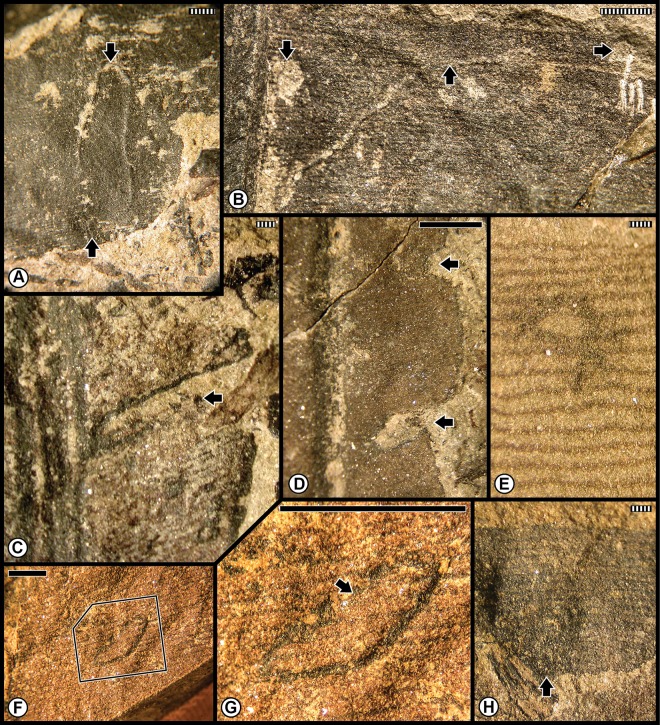
Plant–insect interactions from the Middle Triassic (Anisian) Valle San Lucano Flora, of the Agordo Formation at Valle San Lucano in the central Dolomites. **A**: Surface feeding (DT30) on the cycadophyte “*Pterophyllum*” sp., with a reaction rim indicated by a black arrow. Specimen PAL-1685. **B**: Another specimen of “*Pterophyllum*” sp, but with a thread-like leaf mine (DT71) (central black arrow) originating at upper right, with the probable oviposition mark (right black arrow), and terminating at upper left (left black arrow). The four vertical marks at upper-right are artifacts. Specimen PAL-1688. **C**: An example of trenched margin feeding (DT15) on *Taeniopteris* sp., with white arrow pointing to reaction-rim area. Specimen PAL-1735. **D**: Cuspate margin feeding (DT12, upper arrow) and trenched margin feeding (DT15, bottom arrow) on *Taeniopteris* sp. Specimen PAL-2093. **E**: Interveinal surface feeding (DT11) on *Taeniopteris* sp. Specimen PAL-1853. **F**: A second example of surface feeding (DT30) on the cycadophyte *Taeniopteris* sp. Specimen PAL-1867. **G**: Enlargement of surface feeding indicated by the template at (F), showing a reaction rim (black arrow). **H**: A gall on secondary veins (DT32) of a pinnule of the cycadophyte *Nilssonia neuberi*. Specimen PAL-590. All specimens are reposited in the Museum of Nature South Tyrol (MNS), in Bolzano, Italy. Scale bars: striped = 1 mm; solid = 10 mm.

A third assemblage of Anisian plants is the Valle San Lucano Flora from the Agordo Formation. This local flora is depauperate in plant taxa, and consists of only a few pteridophytes, one pteridosperm, one cycadophyte, two coniferophyte taxa and an indeterminate seed (Table 1; S1 Table). Almost all of the documented damage is external foliage feeding occurring on cycadophytes (Fig 8; S7 Table). Margin feeding is present as trenched margin feeding (DT15) in one example extending almost to the midrib, and cuspate margin feeding (DT12) in various combinations on two specimens of the cycadophyte *Taeniopteris* sp. (Fig 8C and 8D). Surface feeding also is common, consisting of DT30 on the cycadophytes “*Pterophyllum*” (Fig 8A) and *Taeniopteris* sp. (Fig 8F and 8G), and interveinal feeding with a prominent, thick reaction rim (DT11) on a second specimen of *Taeniopteris* (Fig 8E). A major, intriguing aspect of the leaf damage on these cycadophytes is a long, thread-like leaf mine (DT71) on the cycadophyte “*Pterophyllum*” that extends from the pinnule margin and courses rectilinearly to the midrib, where it ends in an ovoidal, chamber-like expansion (Fig 8B). A second endophytic interaction, an ovoidal, bulbous DT32 gall, occurs along the pinnular midsection of the cycadophyte *Nilssonia neuberi*. Many aspects of the external-foliage-feeding and leaf-mine damage on cycadophytes from the Valle San Lucano Flora presages a similar type of damage in Late Triassic (Carnian) floras of Western Europe [14,57,59].

The first flora encountered from the late Middle Triassic (Ladinian) assemblage is the Monte Cernera Flora, from the Aquatona Formation near Cortina. The Monte Cernera Flora is a moderately diverse plant assemblage, consisting of 15 plant-organ species of pteridophytes, cycadophytes and coniferophytes (Table 1; S1 Table). Lycophytes, sphenophytes, pteridosperms and ginkgophytes are absent. As in the Anisian Furkelpass Flora (Fig 7; S6 Table), the Monte Cernera Flora is represented by minimal herbivory (S8 Table). The DT spectrum is typical of a generalized feeding syndrome, and occurs solely on an unidentified pteridophyte and the cycadophyte *Bjuvia* sp. The flora lacks stereotyped patterns of damage that would indicate more specialized feeding modes.

**Fig 9:**
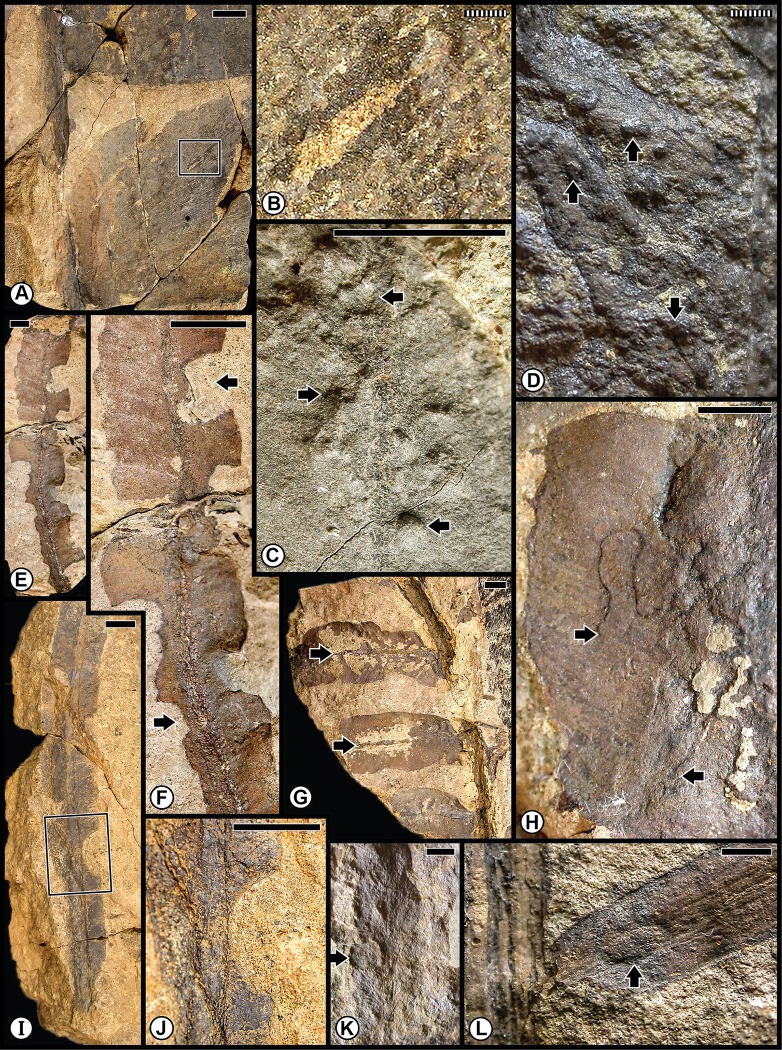
Plant–insect interactions from the Middle Triassic (Ladinian) Monte Agnello Flora, of the Vulcanites near Predazzo in the central Dolomites. **A**: Foliage of the sphenophyte cf. *Schizoneura paradoxa* with slot feeding (DT08). Specimen MGP-194-106. **B**: Enlargement of slot feeding (DT08) indicated in the rectangular template in (A). **C**: The fern *Phlebopteris fiemmensis*, exhibiting DT80 galls. Specimen MGP-181-57C. **D**: Another specimen of *Phlebopteris fiemmensis*, showing small, foliar galls (DT80). Specimen MGP-181-57C. **E**: The pteridosperm *Scytophyllum bergeri* displaying cuspate margin feeding (DT12) along blade edges and margin feeding of the terminus (DT14). Specimen MGP-63-97. **F**: Enlarged magnification of DT12 shown at the left polygon template in (E), illustrating veinal stringers and cuspate excisions (black arrows). **G**: Three pinnules of *Scytophyllum bergeri* showing surface feeding (DT82), the two more prominent examples of which are indicated by white arrows. Specimen MGP-196-39A. **H**: A pinnulular blade of *Scytophyllum bergeri*, showing a thin leaf mine (DT41), with the upper-left arrow showing the mine origin and the lower-right arrow showing the mine probably aborted end. Specimen MGP-63-98A. **I**: An additional specimen of *Scytophyllum bergeri*, displaying examples of margin feeding (DT12, DT13). Specimen MGP-171-28. **J**: An example of DT13 margin feeding. **K**: The possible fern *Speirocarpus* sp., with an example of oviposition (DT101) at left (black arrow). Specimen MGP-197-69B. **L**: An undetermined cycadophyte, showing a midvein gall (DT85). Specimen MGP-196-6. All specimens are reposited in the Museo della Geologia di Predazzo, Italy. Scale bars: striped = 1 mm; solid = 10 mm.

**Fig 10:**
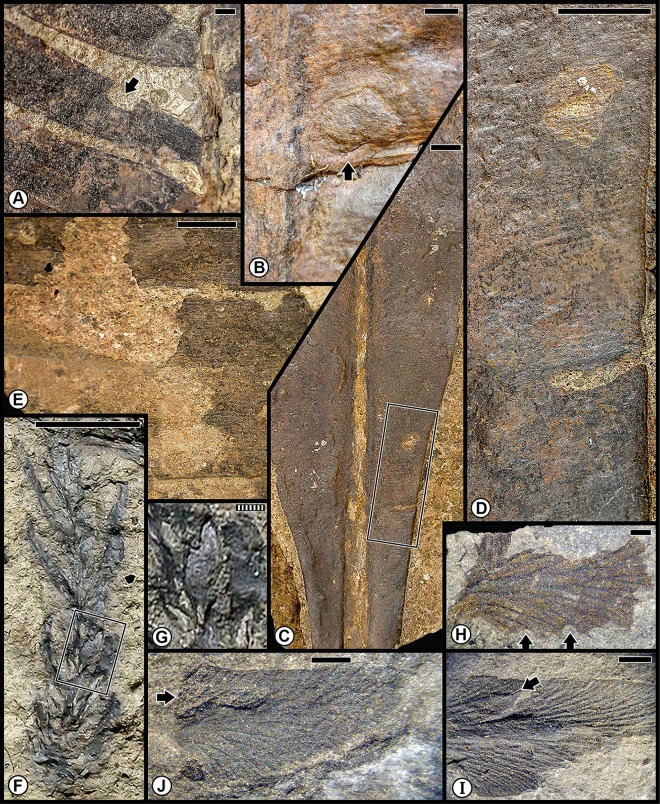
Plant–insect interactions from the Middle Triassic (Ladinian) Monte Agnello Flora, of the Vulcanites near Predazzo in the central Dolomites. **A**: The cycadophyte *Nilssonia neuberi*, displaying cuspate margin feeding (DT12), indicated by the arrow. Specimen MGP-191-6A. **B**: Another example of *Nilssonia neuberi*, exhibiting an example of a scale mark DT77. Specimen MGP-194-72A. **C**: The cycadophyte *Bjuvia* sp., showing examples of surface feeding (DT31) and margin feeding (DT15) along the right margin of the blade. **D**: Enlargement of polylobate surface feeding (DT31) from the upper template and trenched margin feeding (DT15) from the template in (C). **E**: A second example of *Bjuvia* sp., showing successive examples of surface feeding (DT29) on the three lower pinnules. Specimen MGP-196-43. **F**: The coniferophyte *Voltzia* sp., showing a gall indicated in the rectangular template. Specimen MGP-171-81. **G**: Enlargement of the beaked gall (DT121) from the template in (G), showing structure very similar to that described [19] for a walchian conifer. **H**: A *Phlebopteris* sp. pinnule displaying cuspate margin feeding (DT12) along its lower border (arrows). Specimen MPG-191-64. **I**: A *Phlebopteris* sp. pinnule exhibiting a DT106 gall along midveinal area. Specimen MPG-194-84. **J**: A *Phlebopteris* sp. pinnule with a gall at arrow (DT106) occupying the area between the midveinal axis and pinnule edge. Specimen MPG-194-83. All specimens are reposited in the Museo della Geologia di Predazzo, and the Museum of Nature South Tyrol (MNS), Italy. Scale bars: striped = 1 mm; solid = 10 mm.

A second Ladinian assemblage is the Monte Agnello Flora, from the Vulcanites Formation near Predazzo, which contains 18 taxa of lycophytes, sphenophytes, pteridophytes, pteridosperms, cycadophytes, coniferophytes and an unaffiliated taxon. This locality lacks ginkgophytes, as do the other Triassic localities (Table 1; S1 Table). The Monte Agnello Flora exhibits a considerable spectrum of herbivory, such as hole feeding, margin feeding, surface feeding, piercing and sucking and galling on a diverse array of lycophytes, pteridosperms, cycadophytes and coniferophytes (Figs 9 and 10; S9 Table). One of the dominant plant taxa of this flora, the pteridosperm *Scytophyllum bergeri*, has been overwhelmingly targeted by insect herbivores (Fig 9). Examples of insect damage on *S*. *bergeri* include multiple instances of cuspate margin feeding (DT12, DT13, DT14) along pinnule margins, apices and midribs, occasionally with veinal stringers (Fig 9E, 9F, 9I and 9J); surface feeding (DT82) occurring as swaths on both sides of the pinnular blade adjacent to the midrib (Fig 9G); and a thin, serpentine leaf mine (DT41) found on the midsection of a pinnule (Fig 9H). Galls (DT80) were found on the pteridophyte *Phlebopteris fiemmensis* (Fig 9C and 9D); oviposition (DT101) occurs on the putative pteridophyte *Speirocarpus* sp. (Fig 9K); and a midveinal gall (DT85) is on an unidentified cycadophyte (Fig 9L). The DT85 gall is structurally identical to a midveinal gall described on a callipterid pteridosperm from the Cisuralian of Texas [19]. An unidentified species of *Neuropteridium* harbors frequent examples of cuspate margin feeding (DT12) (Fig 10H), but also circular surface feeding (DT31) and elongate mite galls (DT106) present on several specimens (Fig 10I and 10J). Pinnules of the cycadophyte *Nilssonia neuberi* show cuspate margin feeding (DT12) and scale-insect impressions with distinctive encircling necrotic tissue (DT77) that occurs at the base of pinnules (Fig 10A and 10B). Another cycadophyte, *Bjuvia* sp., exhibits trenched margin feeding (DT15) and circular surface feeding (DT31) along the leaf-blade margin (Fig 10C and 10D); a second example of *Bjuvia* sp. (Fig 10E) provides multiple instances of surface feeding (DT29). The coniferophyte *Voltzia* sp. hosts a basally inflated, pimpled, beaked gall (DT121) attached to the woody axis of a branchlet (Fig 10F and 10G). This gall is nearly identical to one described on a walchian conifer from the Cisuralian of Texas [19]. The preservational quality of the Monte Agnello Flora and its insect damage, including similarities of some rare DTs to those of Cisuralian Texas, is exceptional and warrants further investigation [151,152].

**Fig 11:**
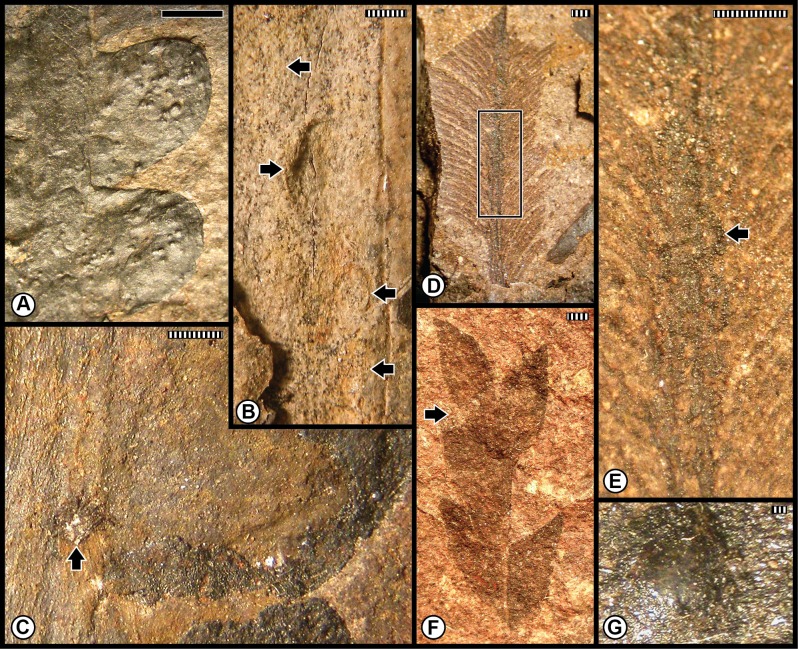
Plant–insect interactions from the Middle Triassic (Ladinian) Forcella da Cians/Ritberg Flora, of the Agordo Formation near Wengen/La Valle, St. Veit-Seewald, within the Fernazza Formation, and St. Veit-Innerkohlbach, near Prags/Braies, within the Wengen/La Valle Formation in the northern Dolomites. **A**: The pteridosperm *Ptilozamites sandbergeri* showing abundant DT80 galls along the leaf surface. Specimen PAL-1516 (St. Veit-Innerkohlbach). **B**: Successive, end-to-end oviposition marks (DT175) on a *Ptilozamites sandbergeri* rachial midvein, showing a series of four lesions (arrows). Specimen PAL-202 (Forcela da Cians). **C**: Oviposition (DT76) on the rachial midvein of another specimen of *Ptilozamites sandbergeri*. Specimen PAL-11 (Forcela da Cians). **D**: A pinnule of *Marattiopsis* sp., showing development of a leaf mine (DT222) adjacent to the primary vein. Specimen PAL-231 (St. Veit-Innerkohlbach). **E**: Enlargement of midvein-centered leaf mine (DT222) in the rectangular template in (D). Arrow shows the mine frass trail along the midrib. **F**: The fern *Cladophlebis leuthardtii*, with a small example of cuspate margin feeding (DT12). Specimen PAL-73 (St. Veit-Innerkohlbach). **G**: Foliar gall (DT32) on a fourth specimen of *Ptilozamites sandbergeri*. Specimen PAL-18 (Forcela da Cians). All specimens are reposited in the Museum of Nature South Tyrol (MNS), in Bolzano, Italy. Scale bars: striped = 1 mm; solid = 10 mm.

The remaining Ladinian assemblages are the Forcella da Cians/Ritberg near Wengen/La Valle, St. Veit-Seewald, within the Fernazza Formation, and St. Veit-Innerkohlbach, near Prags/Braies, within the Wengen/La Valle Formation. These assemblages consist of sphenophytes, pteridophytes, pteridosperms, cycadophytes, coniferophytes and an affiliated seed, but lacks lycophytes and ginkgophytes (Table 1; S1 Table). The herbivory is moderately diverse (Fig 11; S10–S12 Tables), enriched in specialized over generalized interactions, consists of margin feeding, oviposition, galls and leaf mines, and is deployed predominantly on pteridophytes and the pteridosperm *Ptilozamites sandbergeri* (Figs 11A–11C and 10G). The latter hosts numerous, small galls (DT80) occurring parallel to the pinnular secondary venation (Fig 11A). Other specimens of *P*. *sandbergeri* have successive end-to-end oviposition (DT175) on the rachial midvein (Fig 11B). Another example of a broad oviposition mark on the rachial midvein (DT76) occurs on a third specimen of *P*. *sandbergeri* (Fig 11C), similar to a previously illustrated specimen (Fig 6N and 6O). A bulbous gall with anomalous encircling tissue (DT32) occurs on a fourth specimen of *P*. *sandbergeri* (Fig 11G). The marattialean pteridophyte *Marattiopsis* sp. houses a delicate, curvilinear leaf mine (DT222) that courses within the pinnular midrib and exhibits a thickened frass trail (Fig 11D and 11E). The filicalean pteridophyte *Cladophlebis leuthardtii* possesses an example of cuspate margin feeding (DT12) on one of its pinnules (Fig 11F).

### Quantitative patterns of herbivory

The Tregiovo Flora of Kungurian age consisted of 464 censused foliage items, of which 3.6% exhibited insect-herbivore damage (Fig 2). Based on this value of insect herbivory and on the frequency data of the number of DT occurrences, 73.6% consisted of generalized herbivory, defined as all types of exophytic external foliage feeding such as hole, margin and surface feeding and skeletonization; by contrast, specialized herbivory consisted of 26.3%, defined as all other, endophytic, damage represented by piercing and sucking, oviposition, galling, seed predation and wood boring (Fig 2; S3 Table). However, based on the classification scheme of DTs, 28.6% were generalized and 71.4% were specialized, although the total number of DT categories were quite low (N = 7) (Table 1). The opposite pattern between the DT frequency and DT diversity data suggests that the while the greatest number of DT occurrences were generalized, the greatest variety of DT types however involved specialized interactions. The dominant plant-host groups exhibiting the greatest level of DT occurrences were taxa of uncertain affinities, but these probably are attributed overwhelmingly to seed plants, followed in decreasing order by ferns or pteridosperms, then conifers.

The Bletterbach Flora of Wuchiapingian age had 1531 censused foliage items, representing a more than threefold increase in specimens from the older, Kungurian Tregiovo Flora. Nevertheless, this flora exhibited an herbivory level of 1.95%, a major reduction of herbivory by almost one-half from the Tregiovo Flora (Fig 2; S4 Table). However, of this herbivory, 79% consisted of generalized damage and 21% was apportioned to specialized damage—almost the same ratio that was found for the Tregiovo Flora (Fig 2). By contrast, based on the classification of DTs, 31.3% of all DT categories were generalized and 68.7% were specialized, with the total varieties of DTs being significantly higher (N = 16) than the Kungurian Tregiovo data (Table 1). As in the Tregiovo data, the frequency of generalized DTs as being greater than specialized DTs was opposite to the pattern seen in the DT diversity data, which emphasized specialized over generalized DTs (Fig 2). Although numbers of DT occurrences were low, the dominant groups of herbivorized plants were cycadophytes, and secondarily, conifers.

The Agordo, Kühwiesenkopf and Furkelpass floras, represent a pooled Anisian (early Middle Triassic) floral assemblage, amounting to 1324 censused plant specimens, a slight decrease (13.5%) from the earlier, Wuchiapingian Bletterbach Flora. The level of insect damage in the pooled Anisian assemblage was 11.6% overall (Fig 2), representing about a fourfold increase from that of the Bletterbach Flora. About 65.5% of the herbivory is attributable to generalized feeding whereas 34.4% is attributable to specialized modes of feeding (Fig 2), a lessening of the ratio of generalized-to-specialized herbivory in the Bletterbach Flora. However, when evaluated by DT categorization (N = 38), 39.5% of all DTs represent generalized damage whereas 60.5% represented specialized damage (Table 1), again indicating a reversal of the pattern based on DT frequency data. The dominant plant taxa that hosted insect herbivores were pteridosperms and to a lesser extent cycadophytes, followed more distantly in decreasing order by pteridophytes, lycophytes and conifers.

The Monte Cernera, Monte Agnello, Forcela da Cians, St. Veit-Seewald and St. Veit-Innerkohlbach, floras collectively represented a pooled Ladinian assemblage that consisted of 923 specimens, down 30% from the total number of Anisian specimens. The level of damage was 10.73% (Fig 3). Of the total herbivory, 73.2% was generalized and 26.8% was specialized, representing a major increase in generalized herbivory compared to the pooled Anisian value (Fig 2). However, when evaluated by DT categorization (N = 24), 54.2% of all DTs were generalized and 45.8% of DTs were specialized (Table 1), indicating a shift toward approximate equivalence between generalized and specialized interactions when compared to DT frequency data from the other sites. The Monte Agnello Flora registered the greatest amount of herbivory (S9 Table), possibly because sample size was the greatest of any Ladinian flora. The local floras of Seewald and Innerkohlbach exhibited even lower generalization-to-specialization ratios, potentially indicating regional variation in levels of host-plant specialization, although such ratios were based on few specimen numbers. The targeted plant hosts were predominantly insecurely identified pteridophytes or pteridosperms, followed more distantly by pteridosperms, and at minor levels, cycadophytes, pteridophytes and conifers. If some of the most abundant herbivorized Ladinian plant hosts are pteridosperms rather than pteridophytes, then it seems likely that the targeting of Ladinian seed plants by insect herbivores retained the trend of targeting of seed plants that began during the Kungurian (Tregiovo Flora), continuing through the Wuchiapingian (Bletterbach Flora) and into the Anisian.

### Patterns of host specificity

Fifty-four plant-arthropod interactions representing 345 separate DT occurrences were recorded, in ca. 7.0% of the Dolomites samples (Figs 2 and 3). Overall, most of these interactions are exophytic (76.8%); 22.6% are endophytic (Table 2). The commonest associations are two types of external foliage feeding, DT12 and DT14, cuspate and apex margin feeding respectively, that account for 58.1% (154/265) of all the damage. The other commonest external foliage feeding consisted of three types of hole feeding, DT01, DT02 and DT08 that amounted to 13.9% (37/265) of all interactions. Oviposition also was represented by six different damage types, principally as insertion scars on foliage, accounting for 11.7% (31/265) of the total damage. Among the endophytic interactions, borings were rarely found as tunnels in twigs with circular to ellipsoidal cross sections, indicated by DT160 constituting 1.3% (1/78) of the total damage.

**Table 2:** Damage Type (DT) Occurrences on the plant hosts from the early Permian (Kungurian) to Middle Triassic (Ladinian) of northeastern Italy organized by functional feeding groups (FFGs) and subgroups and damage types (DTs).

Plant–insect interactional data for all floras		Lycophytes	Sphenophytes	Pteridophytes	Pteridosperms	Pteridophytes or Pteridosperms	Ginkgophytes	Cycadophytes	Coniferophytes	Incertae sedis		
Functional Feeding Groups	Damage types										Subtotals	Percentages
*Exophytic interaction*												
**Hole Feeding**	DT1	0	0	1	0	0	0	6	5	1	**13**	**3.8**
„	DT2	0	0	3	2	0	3	5	0	0	**13**	**3.8**
„	DT3	1	0	1	3	0	0	1	0	1	**7**	**2.0**
„	DT5	0	0	0	1	0	0	0	0	0	**1**	**0.3**
„	DT7	0	0	0	0	0	0	1	0	0	**1**	**0.3**
„	DT8	1	0	1	3	0	0	5	1	0	**11**	**3.2**
„	DT63	0	0	0	1	0	0	0	0	0	**1**	**0.3**
„	DT113	0	0	0	0	0	0	1	0	0	**1**	**0.3**
**Margin Feeding**	DT12	2	0	14	38	1	5	47	18	8	**133**	**38.6**
„	DT14	0	0	4	12	0	0	5	0	0	**21**	**6.1**
„	DT15	0	0	0	0	0	0	6	0	0	**6**	**1.7**
„	DT13	0	0	0	1	0	0	3	1	0	**5**	**1.4**
„	DT143	0	0	0	2	0	0	2	1	0	**5**	**1.4**
**Skeletonization**	DT17	0	0	0	0	0	0	1	0	0	**1**	**0.3**
**Surface Feeding**	DT30	0	0	0	1	0	1	4	1	0	**7**	**2.0**
„	DT29	0	0	0	0	0	0	2	1	0	**3**	**0.9**
„	DT103	0	0	0	2	0	0	1	0	0	**3**	**0.9**
„	DT201	0	0	0	0	0	0	1	0	0	**1**	**0.3**
„	DT203	0	0	0	0	0	0	1	0	0	**1**	**0.3**
**Oviposition**	DT76	0	0	1	5	0	0	6	0	0	**12**	**3.5**
„	DT72	0	0	0	1	0	0	6	1	0	**8**	**2.3**
„	DT100	0	0	0	0	0	0	2	0	1	**3**	**0.9**
„	DT101	0	0	0	0	0	0	1	2	1	**4**	**1.2**
„	DT102	0	0	0	1	0	0	0	0	0	**1**	**0.3**
„	DT175	0	0	0	3	0	0	0	0	0	**3**	**0.9**
*Endophytic interactions*												
**Galling**	DT11	0	0	0	2	0	0	1	1	0	**4**	**1.2**
„	DT32	0	0	1	2	0	1	5	0	0	**9**	**2.6**
„	DT33	0	0	0	0	0	0	0	1	0	**1**	**0.3**
„	DT80	0	0	1	5	0	1	4	3	0	**14**	**4.1**
„	DT85	1	0	0	0	0	0	0	0	0	**1**	**0.3**
„	DT115	1	0	0	0	0	0	0	0	0	**1**	**0.3**
„	DT119	0	0	0	0	0	0	0	1	0	**1**	**0.3**
„	DT120	0	0	0	0	0	0	1	0	0	**1**	**0.3**
„	DT121	0	0	0	0	0	0	0	7	0	**7**	**2,0**
„	DT145	0	0	0	0	0	0	1	0	0	**1**	**0.3**
„	DT153	0	0	0	0	0	0	0	1	0	**1**	**0.3**
„	DT197	0	0	0	0	0	0	2	0	0	**2**	**0.6**
**Mining**	DT40	0	0	0	1	0	0	0	0	0	**1**	**0.3**
„	DT41	0	0	0	1	0	0	0	0	0	**1**	**0.3**
„	DT69	0	0	0	2	0	0	0	0	0	**2**	**0.6**
„	DT71	0	0	1	1	0	0	2	0	0	**4**	**1.2**
„	DT185	0	0	0	0	0	0	1	0	0	**1**	**0.3**
„	DT222	0	0	1	0	0	0	0	0	0	**1**	**0.3**
**Piercing and sucking**	DT46	0	0	0	2	0	0	1	1	0	**4**	**1.2**
„	DT47	0	0	0	1	0	0	2	1	0	**4**	**1.2**
„	DT48	0	0	0	0	0	0	1	2	0	**3**	**0.9**
„	DT77	2	0	1	0	0	0	0	1	0	**4**	**1.2**
„	DT128	0	0	0	0	0	0	1	0	0	**1**	**0.3**
„	DT132	0	0	0	0	0	0	0	1	0	**1**	**0.3**
„	DT133	0	0	0	0	0	0	0	1	0	**1**	**0.3**
„	DT183	3	0	0	0	0	0	1	1	0	**5**	**1.4**
**Seed predation**	DT74	0	0	0	0	0	0	0	0	1	**1**	**0.3**
**Wood boring**	DT160	0	0	0	0	0	0	0	1	0	**1**	**0.3**
*Pathogenic interactions*												
**Fungal necroses**	DT58	0	0	0	0	0	1	0	1	0	**2**	**0.6**
											**345**	**100**
**Occurrences (#):**		**11**	**0**	**30**	**93**	**1**	**12**	**130**	**55**	**13**	**345**	
**Frequency (%):**		**3,2**	**0**	**8,7**	**27,0**	**0,3**	**3,5**	**37,7**	**15,9**	**3,8**	**100**	
**Functional Feeding Groups:**		**4**	**0**	**6**	**7**	**1**	**5**	**8**	**8**	**4**		
**herbivorized specimens (%):**		**5,45**	**0**	**7,60**	**32,03**	**5,26**	**1,83**	**18,87**	**2,88**	**2,84**		

There is evidence for host specificity among the Dolomites localities. Generally, endophytic interactions were sparse, and DTs associated with leaf mining, seed predation and wood boring were rare; however, a variety of gall DTs were more commonly encountered (Table 2). Although the frequency of generalized associations were significantly greater in each of the four major time intervals examined, the fraction of those DTs attributed to generalized interactions were significantly less than those attributable to specialized interactions, except for the Ladinian pooled floras, in which there was approximate equivalence between generalized and specialized DTs (Table 1). The opposite results between DT frequency and DT diversity data throughout most of the Permian and Triassic interval suggest that, while there was a high frequency of generalized interactions, simultaneously the proportionately fewer specialized interactions were more intensely partitioning host-plant tissues and targeting certain plant hosts (Table 1). Although this disparate pattern between elevated abundance of individual generalized interactions versus the greater diversity of particular specialized interactions occurs throughout the interval, the data suggest that DT diversity during Ladinian times was approximately equally partitioned between generalized DTs and specialized DTs.

### Comparison of Permian and Triassic herbivore component communities

**Fig 12:**
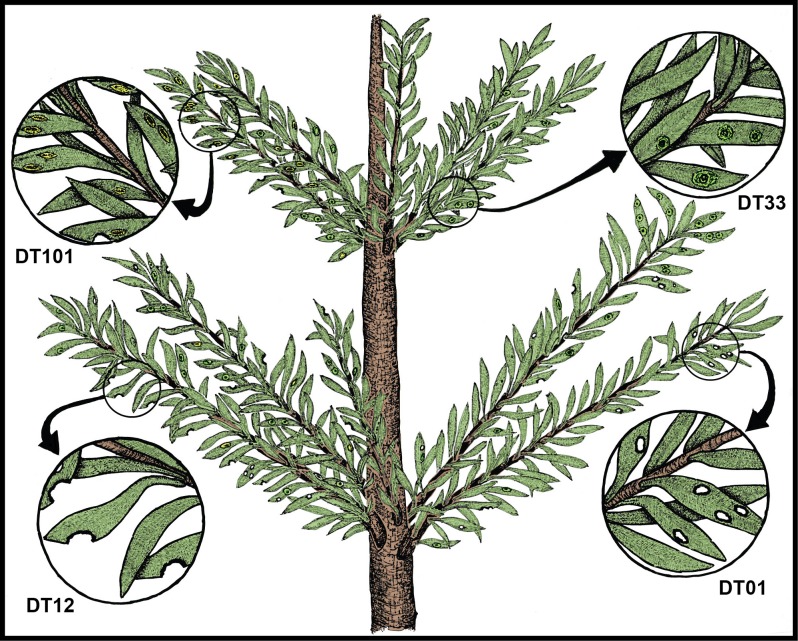
The insect herbivore component community of the conifer *Pseudovoltzia liebeana*, from the Lopingian (Wuchiapingian) Gröden/Val Gardena Flora of northeastern Italy. Four DTs are represented on this host plant, including counterclockwise from the lower right: hole feeding of DT01, margin feeding of DT12, oviposition of DT101, and galling of DT33 (see Fig 5M). Herbivore insect culprits responsible for these DTs likely were beetles (DT01), orthopteroids (DT12), sternorrhynchan hemipterans (DT33) and paleodictyopteroids or polyphagan beetles (DT101) [47,149,150,159]. Possible predators of the insect consumers of these herbivores likely included odonatans, heteropterans, neuropterans and polyphagan beetles.

**Fig 13:**
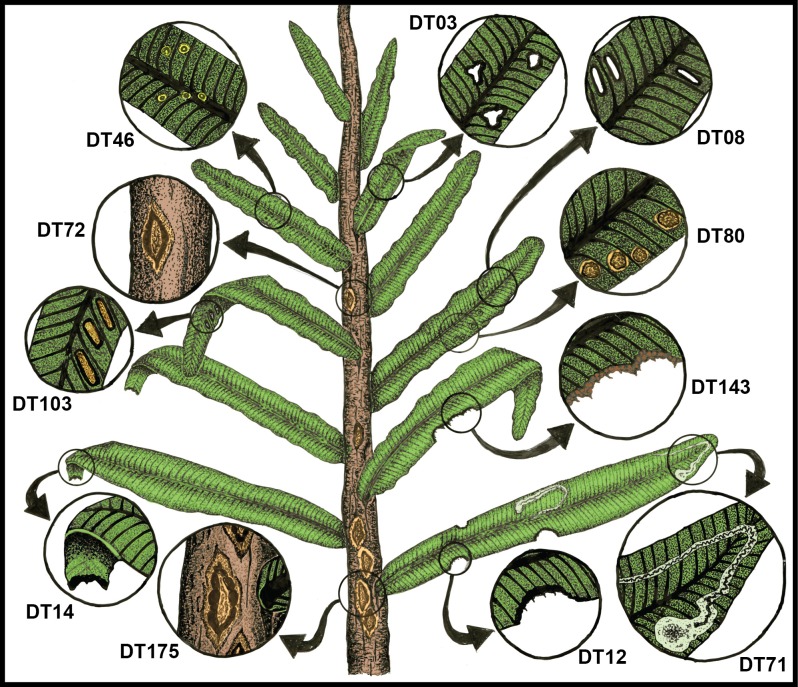
The insect herbivore component community of pteridosperm *Scytophyllum bergeri*, from the Middle Triassic (Anisian) Furkelpass floras of northeastern Italy. Eleven DTs are represented on this host plant, including counterclockwise from the upper left: piercing and sucking of DT46 (Fig 7K); single-egg oviposition of DT72 (Fig 7I); surface feeding of DT103 (Fig 7M), a related DT also recorded as DT82 in Fig 9G; margin feeding on pinnule apices of DT14 (Figs 7A, 7B, 7D, 7J and 9E); multiple-egg oviposition of DT175 (Fig 7L); single pinnular margin feeding of DT12 (Figs 7F, 7J, 9E, 9F and 9I); leaf mining of DT71 (Fig 7N), also shown in Fig 9H as DT41; continuous pinnular margin feeding of DT143 (Fig 7C–7E); galling of DT80 (not Figd); slot hole feeding of DT08 (Fig 7G and 7H); and polylobate hole feeding of DT03 (not Figd). Herbivore insect culprits for these DTs likely were ovipositing odonatans (DT72, DT175), orthopterans (DT12, DT14, DT143), thysanopterans (DT46), sternorrhynchan and heteropteran hemipterans (DT46, DT80), chresmodids (DT72, DT175) tenthredinoid sawflies (DT80, DT143) and polyphagan beetles (DT03, DT08, DT103, DT71) [9,55,57]. Possible predators on the herbivores were odonatans, dermapterans, neuropterans, and adephagan and polyphagan beetles. Note that the herbivore feeding-guild diversity on *S*. *bergeri* is about three times of that on Late Permian *Pseudovoltzia liebeana*.

A comparison between Permian and Triassic plant hosts harboring the most diverse herbivore component community showed major differences in host-specific herbivore patterns before and after the P-Tr event. The Permian plant that displayed the maximum number of DTs was the Wuchiapingian conifer *Pseudovoltzia liebeana* from the Gröden/Val Gardena Sandstone (Fig 12). *Pseudovoltzia liebeana* supported four DTs (DTs 01, 12, 33, 101), of which only one was specialized. By contrast, the Anisian or Ladinian host plant with the greatest number of DTs was the Anisian pteridosperm *Scytophyllum bergeri* from the Furkelpass flora (Fig 13). *Scytophyllum bergeri* had 11 DTs (DTs 03, 08, 12, 14, 46, 71, 72, 80, 103, 143 and 175) that had almost four times the DT diversity and consisted proportionately more endophytic, specialized damage than its Permian conifer counterpart, and included the presence of galls (Figs 9C and 7D) and a leaf mine (Fig 9H).

## Discussion

A worldwide overview of Permian plant–insect interactions ensues in the following discussion, in which there are comparisons of the two Permian floras from northeastern Italy with other floras of Cisuralian and Lopingian age. In particular, an ecological backdrop is reconstructed for the better preserved of these Permian floras, the Wuchiapingian Bletterbach ecosystem. A subsequent and parallel discussion involves the broader, plant–insect interactional context for the Dolomites Anisian and Ladinian floras, focusing on the ecological context that supported the plant–insect interactions of the Monte Agnello ecosystem. A comparison of herbivory and other associations between the Wuchiapingian Bletterbach and the Ladinian Monte Agnello biotas also provides a summary of the principal differences in herbivory styles and the formation of component communities between these two biotas separated by 12 million years and the intervening P-Tr ecological crisis [13,151,152].

### Insect herbivory on Permian plants

The Permian was an interval characterized by an evolutionary and ecological transition from a typical Paleozoic insect fauna centered in the Pennsylvanian (late Carboniferous) to one that increasingly included major lineages that became prominent during the subsequent Mesozoic [6]. For plant–insect interactions, during the Permian there was further herbivore partitioning of the 14 basic tissue types established earlier [10], and evidently greater levels of site-specific richness and intensity of herbivory [17,153]. These increases were matched by the acquisition of unique feeding and ovipositing structures among a wide variety of insects [9,154]. However, the Permian also was important for a style of herbivory that differed significantly from the preceding Pennsylvanian Period consisting of insect lineages involved in an older pattern of herbivory and wood boring centered in wetlands floras [9,10,137,148], supplemented by new modes of external foliage feeding, piercing and sucking, galling, seed predation and oviposition [17,20,42,43,46,55,155–159]. In particular, these associations included external foliage feeding [29,33,36,38‒40,52], dominated by margin feeding; piercing and sucking [17,20,52,154]; oviposition [7,31]; galling [20,31,35,36,159]; seed predation [50]; and wood boring [43,47,149]. Fungal associations, typically associated with herbivory as necrotic tissue [30,32,43,160], were present as well.

In addition to differences in the style of herbivory between earlier plant‒arthropod interactions during the Pennsylvanian, the Permian Period also was characterized by the variety of plant–insect interactions, based on quantitative examinations of bulk floral herbivory. This variability [19,21] includes increased levels in the richness of distinctive DTs as well as the intensity of herbivory in regions such as the Cisuralian of Texas [17‒19,155] in Euramerica, southeastern Brazil [23,26], also see [24] and the Lopingian of South Africa [11]. The Tregiovo Flora of northeastern Italy, also of late Cisuralian age, as is some of the Texan and Brazilian floras, fits into this general pattern of modest but varied herbivory that targeted certain seed-plant taxa that likely was dependent on biogeographical distribution. At Tregiovo, mostly pteridophytes or pteridosperms of uncertain affiliation had the most elevated herbivory levels. By contrast, at the Texan sites, the greatest levels of herbivory were inflicted on gigantopterids at Taint [18], callipterid peltasperms at Coprolite Bone Bed [19] and Colwell Creek Pond [17], and taeniopteroid cycadophytes at Colwell Creek Pond [19] and Mitchell Creek Flats [21]. Herbivory studies from Gondwanan floras, however, indicate that it was glossopterid pteridosperms that was overwhelmingly dominant at sites such as Quitéria, Faxinal, and Morro do Papaléo–Strata 4 and Papaléo–Strata 7/8 [23,26], although glossopterids essentially were the only (and ubiquitous) broadleaved seed plant present in these floras [22,24,25]. The quantitative dominance of herbivory on glossopterids during the Wuchiapingian Clouston Farm Flora of South Africa [11] contrasts floristically with herbivory on a much broader range of similar aged seed-plant groups at the Bletterbach Flora of Northeastern Italy, documented herein.

These examinations of plant–insect interactions in the Cisuralian of Texas, Brazil and the Tregiovo Flora of northeastern Italy of this report, as well as the Lopingian of South Africa [11] and Bletterbach Flora of northeastern Italy described herein, indicate that there is considerable potential for extending quantitative herbivory studies of Permian strata. Permian floras–including Guadalupian age strata which currently lack quantitative analyses–however have been qualitatively described, often as anthropogenically biased evaluations that indicate there are many additional localities that would warrant further quantitative examination. Existing studies frequently describe single plant–arthropod interactions or alternatively a broad variety of selected plant–arthropod interactions such as additional reports from Texas involving galls [20,152] and external foliage feeding [155], and in Europe, from a wide spectrum of Permian localities that mention external foliage feeding, piercing and sucking, oviposition, seed predation and especially wood borings [42,43,46,55,155–159]. For Gondwanan localities, additional, qualitatively based reports originate from: (i) South America, mostly in areas other than Southeastern Brazil describing a variety of feeding damage [16,27,28,161]; (ii) South Africa, centered in the Karoo Basin [9,11,29–32]; (iii) Australia, from several major basins [38–40,160]; (iv) India [33–37,162,163]; and (v) the Palmer Peninsula and outlying islands of Antarctica [149,164,165]. The paleocontinental positions and biogeographic affinities of sites harboring a variety of Artinskian to Changhsingian plant–insect interactions in Cathaysia [49–54] remains poorly known, but evidently represent a full spectrum of plant–insect interactions [9]. These global data suggest that the primary importance of the Bletterbach Locality is that it provides an initial glimpse into a flora from Southern Euramerica that historically has never been examined quantitatively and minimally understood qualitatively.

### Plant–Insect interactions of the Wuchiapingian Bletterbach ecosystem

Most plant material from the Gröden/Val Gardena Sandstone of the Wuchiapingian Bletterbach Flora of northeastern Italy is fragmentary and suboptimally preserved. This preservational condition indicates that plant items were transported parauthchthonously over some distance and consequently did not grow where the plant fossils were deposited. The elevated abundance of seeds and other fructifications suggest that the source area could not have been too distant; otherwise, conifer cones would have been disarticulated, abraded and preserved beyond recognition. Because of the highly fragmentary nature and modest preservational quality of the Bletterbach specimens, the present determination of herbivory level likely represents an underestimation of the actual amount of plant consumption present. Such an under-representation of herbivory would be attributable to unavoidable taphonomic factors [11,52,166], rather than to anthropogenic bias [18,135]. However, the presence of sedimentary charcoal suggests that wildfires occurred nearby or in situ [98,167,168], indicating that the abundance and density of vegetation must have been relatively elevated to provide plant fuel to sustain such fires. Also affecting the preservation of the floras are several paleosol layers displaying variously hued strata that occur within fluvial sedimentary units, indicating redox-associated changes in the sedimentary environment over time. Vertebrates were present at Bletterbach, but only by their footprint ichnofossils that were preserved only under certain sedimentological and other environmental conditions. To date, no skeletal remains have been found.

Within the Bletterbach regional setting of northeastern Italy, the only source of quantitative data on plant–insect interactions from leaf assemblages has been the 3.6% herbivory level from the Kungurian Tregiovo Flora of northeastern Italy [13], and the 1.95% level from the Wuchiapingian Bletterbach Flora (Fig 2, S4 Table) [84,99,100]. Herbivory levels of these two floras are comparable, within a factor of three, to bulk-floral damage values of similar age from paleofloras of Euramerica [17–19,21] and Gondwana [11,23,26]. Despite the thick stratal succession in northeastern Italy, currently recognized, well–preserved and diverse plant fossils are limited to a rather small interval of the exposed section. However, other, currently unexplored floras undoubtedly are present, but would require sedimentological data and knowledge of substrate type to indicate their location. These floras typically have been found within coastal-plain and fluvial deposits.

Insect body fossils were not found at the Bletterbach locality. Nevertheless, there is an ecologically relevant, approximately coeval insect fauna from the Salagou Formation of the Lodève Basin in France [169] and from other, insect–bearing deposits of Late Paleozoic age occurring along the northern and eastern regions of the East European Platform [49]. The Lodève insect fauna is comprised of several orders and about 17 species of insects, including Odonatoptera, Diaphanopterodea, Palaeodictyoptera, orthopteroids and early representatives of modern holometabolous lineages [169], most of which likely were herbivores. By contrast, insect assemblages of the Guadalupian and Lopingian of European Russia are significantly more diverse. To date, these Kungurian to Wordian age insect assemblages consist of 69 families, 81 genera, and 105 species that represent 25 orders of insects [47,147,159], and broadly include diverse orthopteroid groups, and early representatives of several, extant, holometabolous lineages [170]. Based on these results–and taking into account available stratigraphic and plant–insect associational data (Figs 2 and 12)–we tentatively propose that potential insect herbivores at Lodève, European Russia, Bletterbach, and other regionally relevant sites collectively supported a common, diverse insect fauna. A major component of this regional insect fauna was herbivorous taxa as diverse as mandibulate orthopteroids, piercing-and-sucking hemipteroids and the larvae of basal holometabolous lineages. The effects of these varied insect herbivores are present on the component herbivore community on *Pseudovoltzia liebeana*, a broadleaved voltzialean conifer host with a somewhat elevated spectrum of herbivore damage (Fig 12).

### Insect herbivory on Triassic plants

The Triassic interval of the Dolomites from northeastern Italy presents significant differences from the earlier Permian record within this region of Euramerica. Unlike the Lopingian record of the Dolomites, represented by the Bletterbach Flora that contains significant plant–insect associational data, the Early Triassic floral record is virtually absent. Additionally, the Early Triassic record is largely missing from the rest Euramerica as well, representing a five-million-year-long gap for which very little is known of plant–insect interactions. Minor exceptions to this pattern are plant hosts and associated interactions from southeastern Euramerica, in Russia [45,171], also see [147], and the epicontinental, Central European Basin [55]. However, these recent discoveries are uninformative regarding the broader diversity of Early Triassic floras and their insect interactions that would be needed to understand the origin of European Anisian floras. Nevertheless, it appears that during the Early Triassic (Olenekian Stage), there was a major, dynamic expansion of insect herbivory that appears to have been dominated by external foliage feeding but did not exceed the levels reached among end-Permian floras [19,51]. Certainly by Anisian times, the full complement of functional feeding groups was established, with the addition of leaf mining and the significant increase of DTs worldwide (Table 1) [9,151]. This transformation of Middle Triassic floras also is seen in site-specific DT richness in certain major floras [9,14,60,69,172]. To date, one quantitatively based analysis has been made of Triassic plant–insect interactions [152], that together with the current study of other floras from the Dolomites Region, can provide informative comparisons to equivalent studies of the Anisian and early Carnian of South Africa that lately have been only qualitatively examined [9,69]. Features of herbivory such as DT frequency and richness, host specialization and tissue-partitioning patterns in component herbivore communities now can be examined both biogeographically and temporally in the wake of the P-Tr ecological crisis.

There are several regions where qualitative records of Middle to Late Triassic plant–insect interactions have been made that can serve as a basis for understanding quantitatively the effect of the P-Tr event on plant–insect interactions. A variety of interactions have been documented from Euramerican Europe and the southwestern United States; Gondwanan South America, South Africa, India and Australia; and China. For Western Europe, a broad range of interactions have been determined for Anisian to early Carnian floras from Germany, France, Austria and Russia [14,45,57–59,173–176]. The Chinle Formation of the southwestern United States, of Norian age [172,177], has been a second source of Euramerican interaction data that is especially rich in wood borings [60–65,172,178–181]. Several clusters of Gondwanan localities, primarily the Burgersdorp and Molteno formations from South Africa [9,69,155], but also a variety of local basins from South America [67,68], India [181] and Australia [71,182–184], have recorded an array of plant–insect interactions on the dominant hosts of *Heidiphyllum*, *Lepidopteris*, *Dicroidium*, *Sphenobaiera* and *Taeniopteris*. Although only recently explored, China also has provided floras from the North China and South China terranes as a new provenance for Late Triassic plant–insect interactions [66].

### Plant–Insect interactions of the Ladinian Monte Agnello ecosystem

The Monte Agnello Flora of Ladinian age originates from a parautochthonous assemblage of plants occurring within pyroclastic strata. The insect herbivory present in the Monte Agnello flora represents an expansion of DT frequency (12.3%) and richness (19 DTs) that originated in earlier interactions in Dolomites Anisian floras, consisting of DTs attributed to external foliage feeding, piercing and sucking, oviposition, galling and leaf mining (Fig 3; S9 Table) [152]. The dominantly herbivorized host plants are pteridosperms (e.g., *Scytophyllum*), followed subdominantly by cycadophytes (e.g., *Bjuvia*) and pteridophytes (e.g., *Neuropteridium*), and a minor component of conifers (e.g., *Voltzia*) (Figs 11 and 12). *Scytophyllum bergeri* was one of the plant hosts harboring the greatest diversity of DTs for any Ladinian assemblage of the Dolomites (Fig 13), and although the component community of this pteridosperm originates from a spatiotemporally adjacent locality, it probably applies to the current flora as well. Insect interactions of the Monte Agnello Flora indicate (i) an overall increase in the frequency and richness of interactions, (ii) the targeting of seed plants, (iii) subequal representation of generalized and specialized associations, and (iv) the first appearance of leaf mining in the Dolomites Region (Figs 9B and 10H) [152]. The Monte Agnello Flora has the greatest number of plant specimens analyzed in any Ladinian Dolomites assemblage, and likely represents a typical pattern of plant and insect diversity prior to the changes in later Triassic floras of the Molteno Formation in South Africa and Chinle Formation in Arizona.

### Comparisons of herbivory between the Dolomites and other regions

The percentages of leaves damaged (the herbivory index) from the Kungurian Tregiovo Flora and Wuchiapingian Bletterbach Flora of the Dolomites Region are characterized by 3.6% and 1.95%, respectively (Fig 2). These two, herbivory indices of the Permian Dolomites Region, although widely separated in time, mostly are comparable to the Cisuralian Euramerican localities in north-central Texas of Taint (2.58%), Mitchell Creek Flats (1.98%) and Colwell Creek Pond (2.34%), although Coprolite Bone Bed (0.27%) is an outlier [17‒19,21]. Anthropogenic collection bias may have been a minor factor for the Dolomites floras, but would not have affected the Texan floras, as all floral material was bulk collected and not high-graded. However, the two herbivory indices from the Dolomites are significantly lower when compared to the Cisuralian Gondwanan localities in southeastern Brazil of Quitéria (7.41%), Faxinal (7.02%), Morro do Papaléo–Stratum 4 (12.50%) and Morro do Papaléo–Stratum 7/8 (9.42%), which represent an approximate threefold or more increase in Gondwanan herbivory over the Euramerican values. This difference could represent a greater intensity in insect consumption of glossopterid-dominated floras over those of the more diverse, seed-plant floras of Euramerica; or alternatively, may represent a difference in methodology for calculation of herbivory. Although the low values of herbivory at Tregiovo and Bletterbach may be attributable to nonanthropogenic taphonomic factors, there are two substantive patterns that emerge from the plant–insect interaction data.

The first pattern is that varied ectophytic interactions were the dominant pattern of the Permian Tregiovo and Bletterbach Floras. Ectophytic consumption of plants, recorded as external foliage feeding, was a key element of Permian insect herbivore component-community structure (Fig 2) [9,17]. Endophytic consumption of plants, such as piercing and sucking, oviposition, galling and seed predation, was a much lesser element of component-community structure (Fig 2) [9,17]. A second pattern is that only a few, major seed-plant lineages that are variably present in time and space were the most herbivorized group of plants during the Permian [9]. In particular, elevated host specificity occurred on unaffiliated groups and pteridosperms at Tregiovo and on taeniopterids, cycadophytes and conifers at Bletterbach [13,82], versus gigantopterids, callipterid peltasperms and taeniopterids occurring at the three Texan [17–19,21] and glossopterids at the four Brazilian [23,26] localities. The absence of a sufficient number of well-preserved plant specimens at Tregiovo and Bletterbach will require additional, future exploration in the Dolomites and other nearby regions to obtain a better sense of the spectrum of plant‒insect interactions and herbivory levels in Permian Western Europe.

The Middle and Late Triassic floras of the Dolomites recently have become more extensively documented. These Triassic floras represent two major time intervals: (i) the early Middle Triassic (Anisian) of the Dont Formation, such as the Kühwiesenkopf Flora; and (ii) the late Middle Triassic (Ladinian) of the Fernazza and La Valle Formations, including the Ritberg, Seewald and Innerkohlbach floras. For each of these two time intervals, two to four, described plant assemblages represent distinctive habitats as well as a record of lycophytes, sphenophytes, pteridophytes, pteridosperms, cycadophytes, coniferophytes and ginkgophytes (S1 Table). Given the approximate 37 million-year time interval represented by these informative P-Tr floras, the Dolomites should be an ideal setting for examining the effect that the end-Permian event had on plant–insect interactions and the subsequent response from plant hosts, their insect herbivores, and encompassing community structure. The paleoecological and temporal setting of the Italian Dolomites plant–insect interaction record can be compared to that of the South African Karoo Basin of Gondwana [9,69,70]. Such a comparison would contrast the Dolomites of northeastern Italy with the Karoo Basin of Gondwanan South Africa, perhaps providing insight into whether the response of plant hosts and their insect herbivores to the P-Tr event was globally synchronous.

## Conclusions

From this study of floras across the Permian–Triassic interval of the Dolomites (Southern Alps, northeastern Italy), the following six conclusions have been reached. These conclusions provide an initial report on plant–insect relationships before and after the P-Tr event using qualitative and quantitative techniques for evaluating interactions. As such, the conclusions provide a baseline for further study of this event in other regions where there is approximate continuity of plant-bearing strata from the Permian and into the later Triassic.

1. Material examined. The plant–insect interactions were assessed for ten floras from the Permian–Triassic interval of the Dolomites Region of Northeastern Italy: the Kungurian Tregiovo Flora (Cisuralian Permian), the Wuchiapingian Bletterbach Flora (Lopingian Permian), three pooled Anisian floras (early Middle Triassic), and five pooled Ladinian floras (late Middle Triassic). Plant–insect data were based on examination of 4242 plant specimens (1995 Permian, 2247 Triassic) allocated to 86 fossil taxa (32 Permian, 56 Triassic) from lycophytes, sphenophytes, pteridophytes, pteridosperms, coniferophytes, ginkgophytes, cycadophytes and unaffiliated taxa representing a 37 million-year interval (23 m.y. Permian, 14 m.y. Triassic).

2. General pattern of insect herbivory. The percentage of insect-damaged leaves for each bulk flora, as measured by the number of damage type (DT) occurrences, if any, per leaf [134], was: 3.6% (N = 464 leaves) for the Tregiovo Flora, 1.95% (N = 1531 leaves) for the Bletterbach Flora, 11.65% (N = 1324 leaves) for the pooled Anisian flora; and 10.72% (N = 923 leaves) for the pooled Ladinian flora. This documents a rise in herbivory, as measured by the number of leaves with DTs as a fraction of the total number of leaves in a flora.

3. Host-plant generalization versus specialization. Generalized insect damage, defined as external foliage feeding, was 73.6% for the Tregiovo Flora, 79.0% for the Bletterbach Flora, 65.5% for the pooled Anisian flora, and 73.2% for the pooled Ladinian flora. This trend suggests that about three fourths of the incidence of associations were generalized across the studied interval, except for the Anisian, during which approximately 3 out of 8 interactions were specialized. In addition, throughout the interval, although the frequency of occurrences was greatest for generalized DTs on leaves, the much smaller fraction of DTs–about one-fifth–contained a far greater proportion of specialized DTs.

4. Insect herbivore component communities. The host plant bearing the most elevated number of DTs from the Wuchiapingian Bletterbach Flora was *Pseudovoltzia liebeana*, which possessed a component herbivore community of 4 DTs. This component community is compared to the host plant *Scytophyllum bergeri*, from the pooled Ladinian Flora, which possessed 11 DTs, representing an increase of almost three times. These Permian–Wuchiapingian versus Triassic–Ladinian values indicate not only a major rise in herbivory intensity, but more importantly, tissue-level specialization as measured by herbivore component community structure.

5. Possible taphonomic limitations. Low rates of herbivory, such as those from the Permian Tregiovo and Bletterbach floras may be attributed to inadequate preservation. However, the presence a sufficient number of sampled specimens may mitigate any taphonomic restrictions that would reduce the quality of the retrieved data.

6. Comparisons to other localities. The percentage of lower Permian (Cisuralian) insect-damaged leaves between the Dolomites and north-central Texas are approximately the same, whereas those of the Dolomites are approximately one-third that of southeastern Brazil.

## Supporting Information

S1 Table Distribution of early Permian (Kungurian) to Middle Triassic (Ladinian) plant taxa from northeastern Italy. (PDF)

S2 Table Plant-group herbivory levels for the examined floras from the early Permian (Kungurian) to Middle Triassic (Ladinian). As shown in Figs 2 and 3.
(PDF)

S3 Table Insect herbivory of the Tregiovo Flora (Cisuralian). (PDF)

S4 Table Insect herbivory of the Gröden/Val Gardena Flora (Lopingian). (PDF)

S5 Table Insect herbivory of the Kühwiesenkopf/Monte Prà della Vacca Flora of the Dont Formation (Anisian). (PDF)

S6 Table Insect herbivory of the Furkelpass/Passo Furcia Flora of the Richthofen Formation near Olang (Valdaora) (Anisian). (PDF)

S7 Table Insect herbivory of the Valle San Lucano Flora of the Agordo Formation (Anisian). (PDF)

S8 Table Insect herbivory of the plant assemblage at Monte Cernera of the Aquatona Formation (Ladinian). (PDF)

S9 Table Insect herbivory of the Monte Agnello Flora (Ladinian). (PDF)

S10 Table Insect herbivory of Forcella da Cians/Ritberg, near Wengen/La Valle (Ladinian). (PDF)

S11 Table Insect herbivory of St. Veit-Seewald, Fernazza Formation (Ladinian). (PDF)

S12 Table Insect herbivory of St. Veit-Innerkohlbach, near Prags/Braies, Wengen/La Valle Formation (Ladinian). (PDF)
