# Supplementary material for: Floral Assemblages and Patterns of Insect Herbivory during the Permian to Triassic of Northeastern Italy
Source: PLoS One. 2016 Nov 9;11(11):e0165205. doi: 10.1371/journal.pone.0165205 (PMC5102457; doi:10.1371/journal.pone.0165205)
Supplement: S1 Table — (PDF) [file pone.0165205.s001.pdf]

**S1 Table.** Distribution of Early Permian (Kungurian) to Middle Triassic (Ladinian) plant taxa, northeastern Italy.

| | **PERMIAN** | | **TRIASSIC** | | | | | | | | | | |
|---|---|---|---|---|---|---|---|---|---|---|---|---|---|
| | EARLY | LATE | EARLY MIDDLE | | | | | | LATE MIDDLE | | | | |
| | Kun-gurian | Wuchia-pingian | Anisian | | | | | | Ladinian | | | | |
| **PLANT TAXA** | Tregivo | Bletter-bach | Agordo | Kühwie-senkopf | Hochal-penkopf | Maurer-kopf | Monte Rite | Furkel-pass | Cernera | Monte Agnello | Forcela da Cians | St. Veit Seewald | Inner-kohlbach |
| **Lycophytes** | | | | | | | | | | | | | |
| *Isoetites brandneri* | | | | **X** | | | | | | | | | |
| *Lepacyclotes bechstaedtii* | | | | **X** | | | | **X** | | | | | |
| *Lycopia dezanchei* | | | | **X** | | | | | | | | | |
| *Selaginellites leonardii* | | | | **X** | | | | | | | | | |
| **Sphenophytes** | | | | | | | | | | | | | |
| *Annularia* sp. | **X** | | | | | | | | | | | | |
| *Calamites* sp. | **X** | | | | | | | | | | | | |
| Indet. | | **X** | | | | | | | | | | | |
| *Echinostachys* sp. | | | | **X** | | | | | | | | | |
| *Equisetites arenaceus* | | | | | | | | | | | **X** | | |
| *Equisetites mougeotii* | | | | **X** | | **X** | | **X** | | | | | |
| *Neocalamites asperrimus* | | | | **X** | | | | | | | | | |
| *Radicites* sp. | | | | **X** | | | | | **X** | | | | |
| *Schizoneura paradoxa* | | | | | | | | | | **cf** | | | |
| **Pteridophytes** | | | | | | | | | | | | | |
| *Anomopteris mougeotii* | | | | **X** | | | | | **X** | | | | |
| *Chiropteris monteagnellii* | | | | | | | | | | **X** | | | |
| *Cladophlebis ladinica* | | | | | | | | | | **X** | | | |
| *Cladophlebis leuthardtii* | | | | | | | | **X** | **X** | | **X** | | **X** |
| *Cladophlebis remota* | | | **X** | **X** | | | | **X** | | | | | |
| *Cladophlebis* sp. 1 | | | | **X** | | | | **X** | | | | | |
| *Cladophlebis* sp. A | | | | | | | | | | **X** | | | |
| *Danaeopsis angustifolia* | | | | **cf** | | | | | | | | | **X** |
| *Gordonopteris lorigae* | | | | **X** | **X** | | | **X** | **X** | | **X** | | |
| *Marattiopsis* sp. | | | | **X** | | | | | **X** | | | | **X** |
| *Neuropteridium elegans* | | | | **X** | | | | **X** | | **X** | | | |
| *Neuropteridium voltzii* | | | **X** | **X** | | | | **X** | | | | | |
| *Neuropteridium* sp. | | | **X** | **X** | | | | | **X** | | **X** | | |

| | **PERMIAN** | | **TRIASSIC** | | | | | | | | | | |
|---|---|---|---|---|---|---|---|---|---|---|---|---|---|
| | EARLY | LATE | EARLY MIDDLE | | | | | | LATE MIDDLE | | | | |
| | Kun-gurian | Wuchia-pingian | Anisian | | | | | | Ladinian | | | | |
| **PLANT TAXA** | Tregivo | Bletter-bach | Agordo | Kühwie-senkopf | Hochal-penkopf | Maurer-kopf | Monte Rite | Furkel-pass | Cernera | Monte Agnello | Forcela da Cians | St. Veit Seewald | Inner-kohlbach |
| *Phlebopteris fiemmensis* | | | | | | | | | | **X** | | | |
| *Scolopendrites grauvogelii* | | | | **X** | | | | | | | | | |
| *Scolopendrites scolopendrioides* | | | | **X** | | | | | | | | | |
| *Sphenopteris schoenleiniana* | | | | **X** | | | | **X** | | | | | |
| *Scolopendrites* sp. | | | | **X** | | | | **X** | | | | | |
| *Thaumatopteris* sp. | | | | | | | | | | **X** | | | |
| **Pteridosperms** | | | | | | | | | | | | | |
| alethopteroid pinnules | | **X** | | | | | | | | | | | |
| *Germaropteris martinsii* | | **X** | | | | | | | | | | | |
| *Peltaspermum bornemannii* | | | | **X** | **X** | | | **X** | | | | | |
| *Peltaspermum* sp. | **X** | **X** | **X** | | | | | | | | | | |
| *Ptilozamites sandbergeri* | | | | **cf** | | | | | | | **X** | **X** | **X** |
| *Sagenopteris* sp. | | | | **X** | | | | **X** | | | | | |
| *Scytophyllum bergeri* | | | | **X** | | | | **X** | | **X** | | | |
| **Pteridophytes or Pteridosperms** | | | | | | | | | | | | | |
| *Lodevia nicklesii* | **X** | | | | | | | | | | | | |
| *Lugardonia paradoxa* | | | | | **X** | | | | | | | | |
| *Sphenopteris kukukiana* | **X** | | | | | | | | | | | | |
| *Sphenopteris patens* | **X** | | | | | | | | | | | | |
| *Sphenopteris suessii* | | **X** | | | | | | | | | | | |
| *Sphenopteris* sp. 1 | | **X** | | | | | | | | | | | |
| Indeterminate foliage | | | | | **X** | | | | | | | | |
| **Ginkgophytes** | | | | | | | | | | | | | |
| *Baiera digitata* | | **X** | | | | | | | | | | | |
| *Dicranophyllum* sp. | | **X** | | | | | | | | | | | |
| *Esterella gracilis* | **X** | | | | | | | | | | | | |
| *Leptostrobus* sp. | | **X** | | | | | | | | | | | |
| *Sphenobaiera* sp. | **X** | | | | | | | | | | | | |
| *Sphenobaiera* sp. 1 | | **X** | | | | | | | | | | | |
| **Cycadophytes** | | | | | | | | | | | | | |
| "*Pterophyllum*" sp. | | | | **X** | | | | | **X** | | | **X** | |

| | **PERMIAN** | | **TRIASSIC** | | | | | | | | | | |
|---|---|---|---|---|---|---|---|---|---|---|---|---|---|
| | EARLY | LATE | EARLY MIDDLE | | | | | | LATE MIDDLE | | | | |
| | Kun-gurian | Wuchia-pingian | Anisian | | | | | | Ladinian | | | | |
| **PLANT TAXA** | Tregivo | Bletter-bach | Agordo | Kühwie-senkopf | Hochal-penkopf | Maurer-kopf | Monte Rite | Furkel-pass | Cernera | Monte Agnello | Forcela da Cians | St. Veit Seewald | Inner-kohlbach |
| *Apoldia* sp. | | | | | | | | | | **X** | | | |
| *Bjuvia dolomitica* | | | | **X** | | | | **X** | **X** | | **X** | | |
| *Bjuvia* sp. | | | | **X** | | | | | **X** | **X** | | | |
| *Dioonitocarpidium* sp. | | | | **X** | | | | **X** | | | | | |
| *Lesleya* sp. | **X** | | | | | | | | | | | | |
| *Nilssonia neuberi* | | | | **cf** | | | | | | **X** | | | |
| *Sphenozamites wengensis* | | | | | | | | | **X** | | **X** | **X** | |
| *Taeniopteris* sp. | | | **X** | **X** | | | | **X** | | **X** | **X** | **X** | |
| *Taeniopteris* sp. 1 | **X** | | | | | | | | | | | | |
| *Taeniopteris* sp. 2 | **X** | | | | | | | | | | | | |
| *Taeniopteris* sp. A | | **X** | | | | | | | | | | | |
| *Taeniopteris* sp. B | | **X** | | | | | | | | | | | |
| **Coniferophytes** | | | | | | | | | | | | | |
| *Albertia* sp. | | | | **X** | | | | | **X** | | | | |
| *Dolomitia cittertiae* | **X** | **X** | | | | | | | | | | | |
| *Elatocladus* sp. | | | | | | | | | **X** | | | | |
| *Feysia* sp. | **X** | | | | | | | | | | | | |
| *Hermitia geinitzii* | **X** | | | | | | | | | | | | |
| *Hermitia* sp. | **X** | | | | | | | | | | | | |
| *Ortiseia leonardii* | **X** | **X** | | | | | | | | | | | |
| *Ortiseia visscheri* | | **X** | | | | | | | | | | | |
| *Pagiophyllum* sp. | **X** | | | | | | | | | | | | |
| *Pelourdea vogesiaca* | | | | **X** | | | | | | **X** | **X** | **X** | **X** |
| *Pseudovoltzia liebeana* | **X** | **X** | | | | | | | | | | | |
| *Quadrocladus* sp. | **X** | **X** | | | | | | | | | | | |
| *Ullmannia* sp. | **X** | | | | | | | | | | | | |
| *Voltzia dolomitica* | | | | | | | | | **X** | | **X** | **X** | **X** |
| *Voltzia ladinica* | | | | | | | | | | | **X** | **X** | **X** |
| *Voltzia pragsensis* | | | | | | | | | | | **X** | **X** | **X** |
| *Voltzia recubariensis* | | | **X** | **X** | | | | | | | | | |
| *Voltzia walchiaeformis* | | | | **X** | | | | | | | | | |

| | PERMIAN | | TRIASSIC | | | | | | | | | | |
|---|---|---|---|---|---|---|---|---|---|---|---|---|---|
| | EARLY | LATE | EARLY MIDDLE | | | | | | LATE MIDDLE | | | | |
| | Kungurian | Wuchiapingian | Anisian | | | | | | Ladinian | | | | |
| **PLANT TAXA** | Tregivo | Bletterbach | Agordo | Kühwiesenkopf | Hochalpenkopf | Maurerkopf | Monte Rite | Furkelpass | Cernera | Monte Agnello | Forcela da Cians | St. Veit Seewald | Innerkohlbach |
| *Voltzia* sp. | | | **X** | | **X** | | **X** | | **X** | **X** | **X** | **X** | **X** |
| *Voltzia* sp. 1 | | | | **X** | | | | | | | | | |
| **Incertae Sedis** | | | | | | | | | | | | | |
| cordaitalean-type leaves | **X** | | | | | | | | | | | | |
| *Dicranophyllum* sp. | **X** | | | | | | | | | | | | |
| indeterminate foliage 1 | **X** | | | | | | | | | | | | |
| indeterminate seed | | | **X** | **X** | | | | | | **X** | | | |
