# Supplementary material for: Floral Assemblages and Patterns of Insect Herbivory during the Permian to Triassic of Northeastern Italy
Source: PLoS One. 2016 Nov 9;11(11):e0165205. doi: 10.1371/journal.pone.0165205 (PMC5102457; doi:10.1371/journal.pone.0165205)
Supplement: S2 Table — As shown in Figs 2 and 3. (PDF) [file pone.0165205.s002.pdf]

S2 Table. Plant-group herbivory levels for the examined floras from the early Permian (Kungurian) to Middle Triassic (Ladinian), as shown in Figs 2 and 3.

Flora	*percent lyco-phytes*	*percent spheno-phytes*	*percent pterido-phytes*	*percent pterido-phytes/sperms*	*percent pterido-sperms*	*percent conifers*	*percent cycado-phytes*	*percent ginkgo-phytes*	*percent unknown affinities*
**Tregiovo**	**0.0**	**0.0**	**0.0**	**9.09**	**0.0**	**2.58**	**0.0**	**0.0**	**17.14**
**Bletterbach**	**0.0**	**0.0**	**0.0**	**0.0**	**0.0**	**2.19**	**8.33**	**1.85**	**1.39**
**Anisian pooled**	**5.44**	**0.0**	**6.44**	**0.0**	**28.57**	**4.89**	**23.02**	**0.0**	**0.0**
Agordo	0.0	0.0	0.0	0.0	0.0	16.66	85.71	0.0	0.0
Kühwiesenkopf	4.98	0.0	6.59	0.0	27.62	4.67	21.26	0.0	0.0
Furkelpass	100.00	0.0	5.26	0.0	38.46	0.0	33.33	0.0	0.0
**Ladinian pooled**	**0.0**	**0.0**	**14.89**	**0.0**	**45.45**	**2.54**	**15.35**	**0.0**	**2.04**
Cernera	0.0	0.0	14.28	0.0	0.0	0.0	16.67	0.0	0.0
Monte Agnello	0.0	0.0	7.14	0.0	54.55	3.33	15.88	0.0	3.85
Forcella da Cians	0.0	0.0	25.00	0.0	42.11	1.92	0.0	0.0	0.0
Seewald	0.0	0.0	0.0	0.0	9.09	1.45	10.00	0.0	0.0
Innerkohlbach	0.0	0.0	50.00	0.0	33.33	0.0	0.0	0.0	0.0
