# Supplementary material for: Floral Assemblages and Patterns of Insect Herbivory during the Permian to Triassic of Northeastern Italy
Source: PLoS One. 2016 Nov 9;11(11):e0165205. doi: 10.1371/journal.pone.0165205 (PMC5102457; doi:10.1371/journal.pone.0165205)
Supplement: S3 Table — (PDF) [file pone.0165205.s003.pdf]

S3 Table. Insect herbivory of the Tregiovo Flora of the early Permian (Cisuralian).

Taxa/groups, their abundances & percentages	Specimen number	Percent damage	Percent specialized	Percent gallers	Percent miners	Number of DTs	Specialized DTs	Generalized DTs	Intermediate DTs	FFGs
**Sphenophytes** [8, 1.72 %]										
Annularia cf. *spinulosa*	2	0	0	0	0	0	0	0	0	0
Annularia sp.	1	0	0	0	0	0	0	0	0	0
Calamites sp.	5	0	0	0	0	0	0	0	0	0
**Pteridosperms** [5, 1.07 %]										
Peltaspermum sp.	5	0	0	0	0	0	0	0	0	0
**Pteridophytes or Pteridosperms** [11, 2.37 %]										
Lodevia nicklesii	3	0	0	0	0	0	0	0	0	0
Sphenopteris kukukiana	3	0	0	0	0	0	0	0	0	0
Sphenopteris patens	5	0.2	0	0	0	1	0	1	0	1
**Ginkgophytes** [8, 1.72 %]										
Esterella gracilis	3	0	0	0	0	0	0	0	0	0
Sphenobaiera sp.	5	0	0	0	0	0	0	0	0	0
**Cycadophytes** [9, 1.93 %]										
Lesleya sp.	3	0	0	0	0	0	0	0	0	0
Taeniopteris sp. 1	3	0	0	0	0	0	0	0	0	0
Taeniopteris sp. 2	3	0	0	0	0	0	0	0	0	0
**Coniferophytes** [388, 83.62 %]										
Dolomitia cittertiae	1	1	0	0	0	1	0	1	0	1
conifer indet.	70	0.0142	0.0142	0.0142	0	1	1	0	0	1
Dwarf shoot type 1	1	0	0	0	0	0	0	0	0	0
Dwarf shoot type 2	1	0	0	0	0	0	0	0	0	0
Feysia sp.	*126*	0.0238	0.0079	0	0	2	1	1	0	2
Pagiophyllum sp.	7	0	0	0	0	0	0	0	0	0
Pseudovoltzia liebeana	4	0	0	0	0	0	0	0	0	0
Pseudovoltzia sp.	3	0	0	0	0	0	0	0	0	0
Quadrocladus sp.	41	0.0243	0.0243	0	0	1	1	0	0	1
Ullmannia sp.	9	0.1111	0	0	0	1	0	1	0	1
Hermitia geinitzii	33	0.0606	0.0303	0	0	2	1	1	0	2
Hermitia sp.	92	0.0108	0	0	0	1	0	1	0	1

**Incertae sedis** [35, 7.54 %]										
cordaitalean-type of leaves	18	0.0555	0	0	0	1	0	0	1	1
Morphotype 1	17	0.2941	0	0	0	2	0	2	0	2
TOTALS	464	0.0366	0.0086	0.0021	0	7	4	2	1	4
