# Supplementary material for: Floral Assemblages and Patterns of Insect Herbivory during the Permian to Triassic of Northeastern Italy
Source: PLoS One. 2016 Nov 9;11(11):e0165205. doi: 10.1371/journal.pone.0165205 (PMC5102457; doi:10.1371/journal.pone.0165205)
Supplement: S4 Table — (PDF) [file pone.0165205.s004.pdf]

S4 Table. Insect herbivory of the Bletterbach Flora of the late Permian (Lopingian).

Taxa/groups, their abundances & percentages	Specimen number	Percent damage	Percent specialized	Percent gallers	Percent miners	Number of DTs	Specialized DTs	Generalized DTs	Intermediate DTs	FFGs
**Sphenophytes** [4, 0.21 %]										
indet. sp.	4	0	0	0	0	0	0	0	0	0
**Pteridosperms** [19, 1.01 %]										
Germaropteris martinsii	5	0	0	0	0	0	0	0	0	0
Peltaspermum sp.	7	0	0	0	0	0	0	0	0	0
Sphenopteris suessii	4	0	0	0	0	0	0	0	0	0
Sphenopteris sp. 1	3	0	0	0	0	0	0	0	0	0
**Ginkgophytes** [741, 39.37 %]										
Baiera digitata	12	0.8333	0	0	0	1	0	1	0	1
Baiera sp.	1	0	0	0	0	0	0	0	0	0
Dicranophyllum sp.	85	0.0353	0	0	0	2	0	2	0	2
Leptostrobus sp.	9	0	0	0	0	0	0	0	0	0
Sphenobaiera sp.	13	0	0	0	0	0	0	0	0	0
indeterminate ginkgophyte	621	0.0177	0.0032	0.0032	0	6	2	2	0	4
**Cycadophytes** [24, 1.28 %]										
Pterophyllum sp.	2	0	0	0	0	0	0	0	0	0
Taeniopteris sp. A	3	0.3333	0.3333	0	0	2	1	0	1	1
Taeniopteris sp. B	10	0	0	0	0	1	0	0	0	0
Taeniopteris sp.	2	0.5	0	0	0	2	0	0	0	1
indeterminate cycad	7	0	0	0	0	0	0	0	0	0
**Coniferophytes** [497, 26.41 %]										
Ortiseia leonardi	253	0.0079	0	0	0	3	0	1	0	2
Ortiseia visscheri	1	0	0	0	0	0	0	0	0	0
Pseudovoltzia liebeana	59	0.0678	0.0169	0.0169	0	4	1	2	1	4
Quadrocladus sp.	157	0.0191	0.0127	0.0064	0	4	2	1	0	4
conifer 1 indet.	4	0	0	0	0	0	0	0	0	0
conifer 2 indet.	24	0	0	0	0	0	0	0	0	0
**Incertae sedis** [595, 31.62 %]										
cone type 1	58	0	0	0	0	0	0	0	0	0
cone type 2 (♀)	13	0	0	0	0	3	0	0	0	1

cone type 3 (♂)	42	0	0	0	0	0	0	0	0	0
indeterminate foliage	3	0.3333	0.3333	0	0	2	1	0	0	2
seed type 1	27	0	0	0	0	0	0	0	0	0
seed type 2	158	0.0064	0	0	0	0	0	0	0	3
seed type 3	2	0	0	0	0	0	0	0	0	0
seed type 4	7	0	0	0	0	0	0	0	0	0
stems	285	0.0281	0.0035	0	0	5	1	0	0	3
wood indet.	1	1	0	0	0	1	0	0	0	0
TOTALS	1531	0.0196	0.0039	0.0026	0	16	6	8	2	5
