# Supplementary material for: Floral Assemblages and Patterns of Insect Herbivory during the Permian to Triassic of Northeastern Italy
Source: PLoS One. 2016 Nov 9;11(11):e0165205. doi: 10.1371/journal.pone.0165205 (PMC5102457; doi:10.1371/journal.pone.0165205)
Supplement: S5 Table — (PDF) [file pone.0165205.s005.pdf]

S5 Table. Insect herbivory of the Kühwiesenkopf/Monte Prà della Vacca Flora of the Dont Formation from the Middle Triassic (Anisian).

Taxa/groups, their abundances & percentages	Specimen number	Percent damage	Percent specialized	Percent gallers	Percent miners	Number of DTs	Specialized DTs	Generalized DTs	Intermediate DTs	FFGs
**Lycophytes** [201, 15.95 %]										
Isoetites brandneri	57	0.0877	0.0350	0.0350	0	5	1	3	1	4
Lepacyclotes bechstaedtii	8	0	0	0	0	0	0	0	0	0
Lycopia dezanchei	133	0.0375	0.0225	0	0	3	1	2	0	2
Selaginellites leonardii	3	0	0	0	0	0	0	0	0	0
**Sphenophytes** [38, 3.01 %]										
Echinostachys sp.	6	0	0	0	0	0	0	0	0	0
Equisetites mougeotii	28	0	0	0	0	0	0	0	0	0
Neocalamites sp.	4	0	0	0	0	0	0	0	0	0
**Pteridophytes** [276, 21.90 %]										
Anomopteris mougeotii	21	0	0	0	0	0	0	0	0	0
Cladophlebis leuthardtii	2	0	0	0	0	0	0	0	0	0
Cladophlebis remota	20	0	0	0	0	0	0	0	0	0
Cladophlebis sp. 1	13	0.0769	0	0	0	1	0	1	0	1
Fern indet.	3	0	0	0	0	0	0	0	0	0
Gordonopteris lorigae	87	0.0689	0.0114	0	0.0114	4	1	3	0	3
Danaeopsis sp.	5	0.4	0.2	0	0	4	1	3	0	2
Marattiopsis sp.	7	0	0	0	0	0	0	0	0	0
Neuropteridium elegans	27	0.1111	0	0	0	3	0	3	0	2
Neuropteridium sp.	1	0	0	0	0	0	0	0	0	0
Neuropteridium voltzii	27	0.1851	0.0370	0.0370	0	4	1	3	0	3
Scolopendrites sp.	23	0	0	0	0	0	0	0	0	0
Scol. scolopendrioides	32	0.0312	0.0312	0	0	1	1	0	0	1
Sphenopteris schoenleiniana	8	0	0	0	0	0	0	0	0	0
**Pteridosperms** [114, 9.04 %]										
Peltaspermum bornemannii	9	0	0	0	0	0	0	0	0	0
Podozamites sp.	9	0.3333	0.3333	0.1111	0	3	3	0	0	2
Ptilozamites sandbergeri	7	0	0	0	0	0	0	0	0	0
Sagenopteris sp.	8	0	0	0	0	0	0	0	0	0

Scytophyllum bergeri	81	0.3580	0.0864	0.0370	0.0370	15	5	8	2	7
**Pteridophytes or Pteridosperms** [1, 0.07 %]										
Lugardonia paradoxa	5	0	0	0	0	0	0	0	0	0
**Cycadophytes** [292, 23.17 %]										
Bjuvia dolomitica	*5*	0.2	0	0	0	2	0	2	0	2
Bjuvia sp.	130	0.2	0.0615	0.0230	0	12	5	7	0	5
Dioonitocarpidium sp.	45	0.1111	0	0.0222	0	3	1	1	1	2
Nilssonia neuberi	1	1	1	0	0	1	1	0	0	1
Pterophyllum sp.	15	0.1333	0.0666	0	0.0666	2	1	1	0	2
Taeniopteris sp.	13	0.4615	0	0	0	4	0	3	1	2
Taeniopteris sp. 1	39	0.2307	0.1282	0.1025	0.0256	8	4	4	0	5
Taeniopteris sp. 2	44	0.25	0.1363	0.0454	0	6	4	2	0	3
**Coniferophytes** [321, 25.47 %]										
Albertia sp.	29	0.1379	0.0344	0.0344	0	4	1	3	0	3
Pelourdea vogesiaca	6	0.1666	0.1666	0	0	1	1	0	0	1
Voltzia recubariensis	59	0.0169	0.0169	0	0	1	0	1	0	1
Voltzia sp.	18	0.0555	0	0	0	1	1	0	0	1
Voltzia sp. 1	102	0.0294	0.0190	0.0196	0	3	2	1	0	2
Voltzia walchiaeformis	14	0	0	0	0	0	0	0	0	0
cone (♀)	67	0	0	0	0	0	0	0	0	0
cone (♂)	13	0.0769	0	0	0	1	0	0	1	1
conifer wood indet.	13	0.3076	0.0769	0.0769	0	4	1	3	0	4
**Incertae sedis** [13, 1.03 %]										
seed type 1	13	0	0	0	0	0	0	0	0	0
TOTALS	1260	0.1079	0.0357	0.0166	0.004	37	17	16	4	6
