# Supplementary material for: Floral Assemblages and Patterns of Insect Herbivory during the Permian to Triassic of Northeastern Italy
Source: PLoS One. 2016 Nov 9;11(11):e0165205. doi: 10.1371/journal.pone.0165205 (PMC5102457; doi:10.1371/journal.pone.0165205)
Supplement: S6 Table — (PDF) [file pone.0165205.s006.pdf]

S6 Table. Insect herbivory of the Furkelpass/Passo Furcia Flora of the Richthofen Formation near Olang (Valdaora) from the Middle Triassic (Anisian).

Taxa/groups, their abundances & percentages	Specimen number	Percent damage	Percent specialized	Percent gallers	Percent miners	Number of DTs	Specialized DTs	Generalized DTs	Intermediate DTs	FFGs
**Lycophytes** [1, 2.22 %]										
Lycopia dezanchei	1	1	1	0	0	2	1	1	0	2
**Sphenophytes** [3, 6.66 %]										
Equisetites mougeotii	3	0	0	0	0	0	0	0	0	0
**Pteridophytes** [19, 42.22 %]										
Cladophlebis leuthardtii	3	0.3333	0	0	0	1	0	0	0	1
Cladophlebis remota	2	0	0	0	0	0	0	0	0	0
Cladophlebis sp. 1	5	0	0	0	0	0	0	0	0	0
Fern indet.	2	0	0	0	0	0	0	0	0	0
Gordonopteris lorigae	1	0	0	0	0	0	0	0	0	0
Neuropteridium elegans	3	0	0	0	0	0	0	0	0	0
Neuropteridium voltzii	1	0	0	0	0	0	0	0	0	0
Scolopendrites sp.	2	0	0	0	0	0	0	0	0	0
**Pteridosperms** [13, 28.88 %]										
Peltaspermum sp.	2	0	0	0	0	0	0	0	0	0
Sagenopteris sp.	3	0	0	0	0	0	0	0	0	0
Scytophyllum bergeri	7	0.7142	0.4285	0	0.1428	9	4	3	2	4
Sphenopteris sp. 1	1	0	0	0	0	0	0	0	0	0
**Cycadophytes** [9, 20.00 %]										
Bjuvia sp.	2	0.5	0	0	0	1	0	1	0	1
Dioonitocarpidium sp.	1	0	0	0	0	0	0	0	0	0
Taeniopteris sp.	6	0.3333	0	0	0	2	0	1	1	2
TOTALS	45	0.2222	0.0888	0	0.0222	11	4	5	2	4
