# Supplementary material for: Floral Assemblages and Patterns of Insect Herbivory during the Permian to Triassic of Northeastern Italy
Source: PLoS One. 2016 Nov 9;11(11):e0165205. doi: 10.1371/journal.pone.0165205 (PMC5102457; doi:10.1371/journal.pone.0165205)
Supplement: S7 Table — (PDF) [file pone.0165205.s007.pdf]

S7 Table. Insect herbivory of the Valle San Lucano Flora of the Agordo Formation of the Middle Triassic (Anisian).

Taxa/groups, their abundances & percentages	Specimen number	Percent damage	Percent specialized	Percent gallers	Percent miners	Number of DTs	Specialized DTs	Generalized DTs	Intermediate DTs	FFGs
**Pteridophytes** [3, 15.78 %]										
Cladophlebis remota	1	0	0	0	0	0	0	0	0	0
Neuropteridium sp.	1	0	0	0	0	0	0	0	0	0
Neuropteridium voltzii	1	0	0	0	0	0	0	0	0	0
**Pteridosperms** [1, 5.26 %]										
Peltaspermum sp.	1	0	0	0	0	0	0	0	0	0
**Cycadophytes** [7, 36.84 %]										
Nilssonia neuberi	1	1	1	1	0	1	1	0	0	1
"Pterophyllum" sp.	3	0.6666	0.3333	0	0.3333	2	1	1	0	2
Taeniopteris sp.	3	1	0.3333	0	0	3	1	2	0	2
**Coniferophytes** [6, 31.58 %]										
Voltzia recubariensis	2	0	0	0	0	0	0	0	0	0
Voltzia sp.	4	0.25	0	0	0	1	0	1	0	1
**Incertae Sedis** [2, 10.52 %]										
seed indet.	2	0	0	0	0	0	0	0	0	0
TOTALS	19	0.3684	0.1578	0.0526	0	5	3	2	0	4
