# Supplementary material for: Floral Assemblages and Patterns of Insect Herbivory during the Permian to Triassic of Northeastern Italy
Source: PLoS One. 2016 Nov 9;11(11):e0165205. doi: 10.1371/journal.pone.0165205 (PMC5102457; doi:10.1371/journal.pone.0165205)
Supplement: S8 Table — (PDF) [file pone.0165205.s008.pdf]

S8 Table. Insect herbivory of the plant assemblage at Monte Cernera of the Aquatona Formation of the Middle Triassic (Ladinian).

Taxa/groups, their abundances & percentages	Specimen number	Percent damage	Percent specialized	Percent gallers	Percent miners	Number of DTs	Specialized DTs	Generalized DTs	Intermediate DTs	FFGs
**Pteridophytes** [7, 24.13 %]										
Anomopteris mougeotii	1	0	0	0	0	0	0	0	0	0
Cladophlebis leuthardtii	1	0	0	0	0	0	0	0	0	0
Gordonopteris lorigae	1	0	0	0	0	0	0	0	0	0
Marattiopsis sp.	1	0	0	0	0	0	0	0	0	0
Neuropteridium sp.	2	0	0	0	0	0	0	0	0	0
Pteridophyta indet.	1	1	0	0	0	1	0	1	0	1
**Cycadophytes** [6, 20.68 %]										
Bjuvia dolomitica	1	0	0	0	0	0	0	0	0	0
Bjuvia sp.	3	0.3333	0	0	0	1	0	1	0	1
"Pterophyllum" sp.	1	0	0	0	0	0	0	0	0	0
Sphenozamites wengensis	1	0	0	0	0	0	0	0	0	0
**Coniferophytes** [14, 48.27 %]										
Albertia sp.	1	0	0	0	0	0	0	0	0	0
Elatocladus sp.	1	0	0	0	0	0	0	0	0	0
Voltzia dolomitica	6	0	0	0	0	0	0	0	0	0
Voltzia sp.	6	0	0	0	0	0	0	0	0	0
**Incertae Sedis** [2, 6.89 %]										
seed indet.	2	0	0	0	0	0	0	0	0	0
TOTALS	29	0.0689	0	0	0	1	0	1	0	1
