# Supplementary material for: Floral Assemblages and Patterns of Insect Herbivory during the Permian to Triassic of Northeastern Italy
Source: PLoS One. 2016 Nov 9;11(11):e0165205. doi: 10.1371/journal.pone.0165205 (PMC5102457; doi:10.1371/journal.pone.0165205)
Supplement: S9 Table — (PDF) [file pone.0165205.s009.pdf]

S9 Table. Insect herbivory of the Monte Agnello Flora of the Middle Triassic (Ladinian) from the Dolomites Region of northeastern Italy.

Taxa/groups, their abundances & percentages	Specimen number	Percent damage	Percent specialized	Percent gallers	Percent miners	Number of DTs	Specialized DTs	Generalized DTs	Intermediate DTs	FFGs
**Sphenophytes** [7, 1.07 %]										
Radicites sp.	1	0	0	0	0	0	0	0	0	0
Schizoneura paradoxa	6	0	0	0	0	0	0	0	0	0
**Pteridophytes** [28, 8.47 %]										
Chiropteris monteagnellii	12	0	0	0	0	0	0	0	0	0
Cladophlebis ladinica	4	0	0	0	0	0	0	0	0	0
Cladophlebis sp. A	2	0	0	0	0	0	0	0	0	0
Neuropteridium elegans	3	0	0	0	0	0	0	0	0	0
Phlebopteris fiemmensis	6	0.3333	0.1667	0.1677	0	4	3	1	0	2
Thaumopteris sp.	1	0	0	0	0	0	0	0	0	0
**Pteridosperms** [55, 25.88 %]										
Scytophyllum bergeri	55	0.5455	0.0727	0.0364	0.0181	8	3	6	1	4
**Cycadophytes** [233, 35.90 %]										
"*Pterophyllum*" sp.	1	0	0	0	0	0	0	0	0	0
Apoldia sp.	37	0.1351	0.0270	0	0	3	1	1	0	2
Bjuvia sp.	113	0.1592	0.0088	0.0088	0	9	1	6	2	5
Nilssonia neuberi	74	0.1621	0.0135	0	0	6	1	5	0	3
Taeniopteris sp.	8	0.25	0	0	0	2	0	0	0	1
**Coniferophytes** [300, 46.22 %]										
Elatocladus sp.	1	0	0	0	0	0	0	0	0	0
Pelourdea vogesiaca	4	1	0	0	0	1	0	1	0	1
Voltzia sp.	295	0.0305	0.0033	0.0237	0	3	1	1	1	3
**Incertae Sedis** [26, 4.00 %]										
Speirocarpus sp.	24	0.0417	0	0	0	1	1	0	0	1
seed indet.	2	0	0	0	0	0	0	0	0	0
TOTALS	649	0.1232	0.0138	0.0169	0.0015	19	6	11	2	7
