# Supplementary material for: Floral Assemblages and Patterns of Insect Herbivory during the Permian to Triassic of Northeastern Italy
Source: PLoS One. 2016 Nov 9;11(11):e0165205. doi: 10.1371/journal.pone.0165205 (PMC5102457; doi:10.1371/journal.pone.0165205)
Supplement: S10 Table — (PDF) [file pone.0165205.s010.pdf]

S10 Table. Insect herbivory of Forcella da Cians/Ritberg, near Wengen/La Valle of the Middle Triassic (Ladinian) from the Dolomites Region of northeastern Italy.

Taxa/groups, their abundances & percentages	Specimen number	Percent damage	Percent specialized	Percent gallers	Percent miners	Number of DTs	Specialized DTs	Generalized DTs	Intermediate DTs	FFGs
**Sphenophytes** [6, 5.60 %]										
Equisetites arenaceus	6	0	0	0	0	0	0	0	0	0
**Pteridophytes** [8, 7.47 %]										
Cladophlebis leuthardtii	2	0	0	0	0	0	0	0	0	0
Gordonopteris lorigae	2	0	0	0	0	0	0	0	0	0
Neuropteridium sp.	3	0.3333	0	0	0	1	0	1	0	1
Pteridophyta indet.	1	1	0	0	0	1	0	0	1	1
**Pteridosperms** [19, 17.75 %]										
Ptilozamites sandbergeri	19	0.4210	0.1052	0.1052	0	5	2	0	3	2
**Cycadophytes** [5, 4.67 %]										
Bjuvia dolomitica	2	0	0	0	0	0	0	0	0	0
Sphenozamites wengensis	2	0	0	0	0	0	0	0	0	0
Taeniopteris sp.	2	0	0	0	0	0	0	0	0	0
**Coniferophytes** [52, 48.59 %]										
Pelourdea vogesiaca	3	0.3333	0	0	0	1	0	1	0	1
Voltzia dolomitica	13	0	0	0	0	0	0	0	0	0
Voltzia ladinica	12	0	0	0	0	0	0	0	0	0
Voltzia pragsensis	4	0	0	0	0	0	0	0	0	0
Voltzia sp.	12	0	0	0	0	0	0	0	0	0
conifer wood indet.	8	0	0	0	0	0	0	0	0	0
**Incertae Sedis** [17, 15.88 %]										
seed indet.	17	0	0	0	0	0	0	0	0	0
TOTALS	107	0.1028	0.0186	0.0186	0	6	2	1	3	3
