# Supplementary material for: Floral Assemblages and Patterns of Insect Herbivory during the Permian to Triassic of Northeastern Italy
Source: PLoS One. 2016 Nov 9;11(11):e0165205. doi: 10.1371/journal.pone.0165205 (PMC5102457; doi:10.1371/journal.pone.0165205)
Supplement: S11 Table — (PDF) [file pone.0165205.s011.pdf]

S11 Table. Insect herbivory of St. Veit-Seewald, Fernazza Formation of the Middle Triassic (Ladinian) from the Dolomites Region of northeastern Italy.

Taxa/groups, their abundances & percentages	Specimen number	Percent damage	Percent specialized	Percent gallers	Percent miners	Number of DTs	Specialized DTs	Generalized DTs	Intermediate DTs	FFGs
**Pteridosperms** [11, 11.95 %]										
Ptilozamites sandbergeri	11	0.0909	0.0909	0.0909	0	1	1	0	0	1
**Cycadophytes** [10, 10.86 %]										
“*Pterophyllum*“ sp.	1	0	0	0	0	0	1	0	0	0
Sphenozamites wengensis	2	0.5	0.5	0.5	0	2	1	0	1	2
Taeniopteris sp.	7	0	0	0	0	0	1	0	0	0
**Coniferophytes** [69, 75.00 %]										
Pelourdea vogesiaca	17	0.0588	0	0	0	1	0	1	0	1
Voltzia dolomitica	31	0	0	0	0	0	0	0	0	0
Voltzia ladinica	3	0	0	0	0	0	0	0	0	0
Voltzia pragsensis	3	0	0	0	0	0	0	0	0	0
Voltzia sp.	13	0	0	0	0	0	0	0	0	0
conifer wood indet.	2	0	0	0	0	0	0	0	0	0
**Incertae Sedis** [2, 2.08 %]										
seed indet.	2	0	0	0	0	0	0	0	0	0
TOTALS	92	0.0326	0.0217	0.0217	0	4	2	1	1	3
