# Supplementary material for: Floral Assemblages and Patterns of Insect Herbivory during the Permian to Triassic of Northeastern Italy
Source: PLoS One. 2016 Nov 9;11(11):e0165205. doi: 10.1371/journal.pone.0165205 (PMC5102457; doi:10.1371/journal.pone.0165205)
Supplement: S12 Table — (PDF) [file pone.0165205.s012.pdf]

S12 Table. Insect herbivory of St. Veit-Innerkohlbach, near Prags/Braies, Wengen/La Valle Formation of the Middle Triassic (Ladinian) from the Dolomites Region of northeastern Italy.

Taxa/groups, their abundances & percentages	Specimen number	Percent damage	Percent specialized	Percent gallers	Percent miners	Number of DTs	Specialized DTs	Generalized DTs	Intermediate DTs	FFGs
**Pteridophytes** [4, 8.69 %]										
Cladophlebis leuthardtii	2	0.5	0	0	0	1	0	1	0	1
Marattiopsis sp.	2	0.5	0	0	0.5	1	1	0	0	1
**Coniferophytes** [37, 80.43 %]										
Pelourdea vogesiaca	5	0	0	0	0	0	0	0	0	0
Voltzia dolomitica	15	0	0	0	0	0	0	0	0	0
Voltzia ladinica	5	0	0	0	0	0	0	0	0	0
Voltzia pragsensis	3	0	0	0	0	0	0	0	0	0
Voltzia sp.	2	0	0	0	0	0	0	0	0	0
conifer wood indet.	7	0	0	0	0	0	0	0	0	0
**Pteridosperms** [3, 6.52 %]										
Ptilozamites sandbergeri	3	0.3333	0.3333	0.3333	0	1	1	0	0	1
**Incertae Sedis** [2, 4.34 %]										
seed indet.	2	0	0	0	0	0	0	0	0	0
TOTALS	46	0.0652	0.0434	0.0217	0.0217	3	2	1	0	3
